# Patient-derived organoids reflect the genetic profile of endometrial tumors and predict patient prognosis

**DOI:** 10.1038/s43856-021-00019-x

**Published:** 2021-07-30

**Authors:** Hege F. Berg, Marta Espevold Hjelmeland, Hilde Lien, Heidi Espedal, Tina Fonnes, Aashish Srivastava, Tomasz Stokowy, Elin Strand, Olivera Bozickovic, Ingunn M. Stefansson, Line Bjørge, Jone Trovik, Ingfrid S. Haldorsen, Erling A. Hoivik, Camilla Krakstad

**Affiliations:** 1 Centre for Cancer Biomarkers, Department of Clinical Science, UiB, Bergen, Norway; 2 Department of Gynecology and Obstetrics, Haukeland University Hospital, Bergen, Norway; 3 Section of Radiology, Department of Clinical Medicine, UiB, Bergen, Norway; 4 Mohn Medical Imaging and Visualization Centre, Department of Radiology, Haukeland University Hospital, Bergen, Norway; 5 Section of Bioinformatics, Clinical Laboratory, Haukeland University Hospital, Bergen, Norway; 6 Genomics Core Facility, Department of Clinical Science, UiB, Bergen, Norway; 7 Department of Clinical Medicine, Section for Pathology, Haukeland University Hospital, Bergen, Norway

**Keywords:** Endometrial cancer, Cancer genomics, Chemotherapy, Cancer models, Cancer models

## Abstract

**Background:**

A major hurdle in translational endometrial cancer (EC) research is the lack of robust preclinical models that capture both inter- and intra-tumor heterogeneity. This has hampered the development of new treatment strategies for people with EC.

**Methods:**

EC organoids were derived from resected patient tumor tissue and expanded in a chemically defined medium. Established EC organoids were orthotopically implanted into female NSG mice. Patient tissue and corresponding models were characterized by morphological evaluation, biomarker and gene expression and by whole exome sequencing. A gene signature was defined and its prognostic value was assessed in multiple EC cohorts using Mantel-Cox (log-rank) test. Response to carboplatin and/or paclitaxel was measured in vitro and evaluated in vivo. Statistical difference between groups was calculated using paired t-test.

**Results:**

We report EC organoids established from EC patient tissue, and orthotopic organoid-based patient-derived xenograft models (O-PDXs). The EC organoids and O-PDX models mimic the tissue architecture, protein biomarker expression and genetic profile of the original tissue. Organoids show heterogenous sensitivity to conventional chemotherapy, and drug response is reproduced in vivo. The relevance of these models is further supported by the identification of an organoid-derived prognostic gene signature. This signature is validated as prognostic both in our local patient cohorts and in the TCGA endometrial cancer cohort.

**Conclusions:**

We establish robust model systems that capture both the diversity of endometrial tumors and intra-tumor heterogeneity. These models are highly relevant preclinical tools for the elucidation of the molecular pathogenesis of EC and identification of potential treatment strategies.

## Plain language summary

To study the biology of cancer and test new potential treatments, it is important to use models that mimic patients’ tumors. Such models have largely been lacking in endometrial cancer. We therefore aimed to developing miniature tumors, called “organoids”, directly from patient tumor tissue. Our organoids maintained the characteristics and genetic features of the tumors from which they were derived, would grow into endometrial tumors in mice, and exhibited patient-specific responses to chemotherapy drugs. In summary, we have developed models that will help us better understand the biology of endometrial tumors and can be used to potentially identify new effective drugs for endometrial cancer patients.

## Abstract (web summary)

Berg et al. establish a panel of patient-derived endometrial cancer organoids and xenograft models. They show that their models recapitulate the genetic profile of the donor tumor and can be used for drug testing and development of a prognostic gene signature.

## Introduction

Endometrial cancer (EC) is the most common malignancy of the female reproductive system and the incidence is rising^1^. About 80% of EC cases are classified histologically as endometrioid type (EEC), which is further subcategorized based on architectural grade. Non-endometrioid types comprise the remaining 20%, including serous carcinoma (SC), clear cell carcinoma (CC), and carcinosarcoma^2^. Additionally, four prognostic molecular subgroups have been identified based on genomic abnormalities: *POLE* ultramutated, microsatellite instability hypermutated, copy-number low, and copy-number high^3^. Briefly, most non-endometrioid tumors as well as a smaller fraction of endometrioid tumors are copy-number high cases, while most endometrioid cases typically belong to one of the three remaining molecular subgroups. Molecular classification is now on the verge of clinical implementation initiated by the development of more simplified and cost-effective classification tools^4^.

The first-line treatment of EC is the surgical removal of the uterus and, for patients with high risk or advanced disease, adjuvant chemotherapy with Carboplatin-Paclitaxel and/or radiation^5^. Still, between 15 and 20% of patients experience recurrence^6,7^. Few treatment options are available for this patient group, which is partly explained by the lack of robust preclinical models that mimic key characteristics of endometrial tumors^8,9^. 2D cell cultures are clonally homogenous and genetically unstable^10^, and the molecular spectrum of endometrial tumors has not been captured with the few EC cell lines available^11^. Genetically engineered mouse (GEM) models fail to recapitulate clinical EC disease^12^, and heterotopic patient-derived xenograft (PDX) models poorly mimic the tumor microenvironment and rarely metastasize^13^. Efforts have been made by us and others^14–18^ to successfully generate orthotopic endometrial cancer mouse models. However, this is time consuming, costly, and less suited for high-throughput drug screening and such models also have challenges linked to disease monitoring.

Organoids have recently been developed as in vitro models and show great promise in cancer research^19^. They recapitulate histologic and molecular features of donor tissue, mimic intra- and inter-tumor heterogeneity^20–22^ and emerging studies now demonstrate a matched drug response between the organoids and the corresponding patients^23–26^. Organoids can be engrafted in mice to generate PDX models. Orthotopic implantation is preferred as this provides the cancer cells with a more natural microenvironment, which may affect the tumor growth and disease progression. Invasive and metastatic growth is also frequently observed in orthotopic models, an important strength that allows translational research to explore treatment options for metastatic diseases^13^. A research platform combining organoids with orthotopic organoid-based PDX (O-PDX) models would increase the predictive value of preclinical drug studies by enabling high-throughput testing in vitro based on molecular subtypes with subsequent testing of systemic effects in corresponding in vivo models.

To date, the number of genetically characterized EC organoids is low^27,28^. Also, orthotopic EC PDX models are few^29^, and fully characterized organoid-based orthotopic PDXs are lacking.

Our aim was to establish and demonstrate the robustness and clinical relevance of an organoid-based preclinical platform for EC. We have successfully generated EC organoids from all grades and histological types, including models for hormone receptor (HR) positive low-grade EECs, as well as more aggressive subtypes. Genetic characterization identified known and common genetic alterations in these models, highlighting the relevance of EC organoid models. The organoids were well suited for drug testing and for orthotopic implantation into mice to further study systemic drug effects. Importantly, RNA sequencing reveals that these models recapitulate the relative aggressiveness of endometrial cancer patients and can provide clues about patient prognosis. These models will ultimately improve fundamental and preclinical endometrial cancer research.

## Methods

### Human tissue

Fresh tumor tissue was prospectively obtained from patients with malignant endometrial disease at Haukeland University Hospital, Bergen, Norway. Clinical data and histopathological characteristics were retrieved from patient records and routine pathology reports. The study was approved by the Norwegian regional committees for medical and health research ethics (REK 2014/1907, REK 2018/594). All included patients gave written informed consent.

### Organoid cultures and nomenclature

Fresh tumor biopsies from hysterectomy specimens were kept on ice for transport before incubation in preheated (37 °C) wash medium DMEM/F12 with L-glutamine, Penicillin-Streptomycin, and HEPES (all Gibco) for 20 min. The tissue was minced using a scalpel, followed by digestion with 1.25 U/ml Dispase II (Sigma Aldrich, D4693) and 0.4 mg/ml Collagenase from *Clostridium histolyticum* (Sigma Aldrich, C9263) for 5–30 min at 37 °C. The digested tissue was sampled every 10 min to check for free aggregates of cells. A 100 μm cell strainer was used to separate undigested tissue from aggregated and single cells, which were subsequently washed with red blood cell lysis buffer (Merck). The cells were resuspended in growth factor reduced (GFR) Matrigel (Corning) in a 1:2 volume ratio and seeded in 25 μl droplets in nontreated 48-well plates (VWR). The organoid:Matrigel suspension was solidified at 37 °C and 5% CO_2_ for 20 min before covering each well with 250 μl of modified expansion medium: wash medium supplemented with B27 supplement minus vitamin A (Gibco), recombinant human EGF 100 ng/ml (Peprotec), recombinant human Noggin 100 ng/ml (Peprotec), Y-27632 10 μM (Merck), 17-β estradiol 10 nM (Sigma Aldrich), SB202190 100 nM (Sigma Aldrich), Nicotinamide 10 μM (Sigma Aldrich), A83-01 500 nM (Sigma Aldrich), Recombinant human R-spondin 250 ng/ml (MiltenyiBiotec), and N-acetyl-L-cyteine 1.25 mM (Sigma Aldrich).

The organoids were passaged every 1–2 weeks, depending on the proliferation rate. Passaging was performed by dissolving the organoids by repeatedly pipetting up and down before re-embedding into GFR Matrigel (Corning). Established organoids were stored in our biobank using an expansion medium with 10% DMSO in N_2_(l). Medium withdrawal experiment was performed by seeding organoids as small cell aggregates prior to growth in seven different medium conditions by removal of R-spondin, A83-01, p38 inhibitor, and/or ROCK inhibitor (C1–C9). The number of viable organoids (≥2 cells in cluster) was counted in three replicate wells at Day 1 (24 h after seeding) and at Day 8.

The organoids were considered as O-early at passage <10 and considered O-late at passage >20. Nomenclature for the established organoid cultures was based on organoid/number/grade for endometrioid cultures (OEC-XX-G1/2/3) or organoid/number/type for serous, clear cell, and carcinosarcoma cultures (OEC-XX-SC/CC/CS). Cultures that contain organoids of more than one histological type are indicated with the respective subtype (e.g., E/CC/N), E for endometrioid and N for normal-like organoids (Table 1). The primary tumor donor tissue is indicated with EC (EC-number-grade).

**Table 1:** Established endometrial cancer organoids (*n* = 21).

Organoid ID	Histologic subtype	Histologic grade	FIGO stage	Patient adjuvant therapy	Last status (months follow-up)
OEC-12-G1	Endometrioid	Grade 1	1 A	No	Alive and well (17)
OEC-05-G1/N	Endometrioid	Grade 1	1 A	No	Alive and well (24)
OEC-08-G1	Endometrioid	Grade 1	4B	Carbo + PXL	Dead from disease (11)
OEC-02-G1	Endometrioid	Grade 1	1 A	No	Alive and well (30)
OEC-06-G2	Endometrioid	Grade 2	3C2	Carbo + PXL	Dead from disease (23)
OEC-18-G2	Endometrioid	Grade 2	1 A	No	Relapsed (15)^b^
OEC-20-G2	Endometrioid	Grade 2	1 A	No	Alive and well (-)^c^
OEC-23-G2	Endometrioid	Grade 2	1 A	No	Alive and well (1)^c^
OEC-11-G3	Endometrioid	Grade 3	1B	Carbo + PXL	Alive and well (11)
OEC-03-G3/N	Endometrioid	Grade 3	3 A	Carbo + PXL	Relapsed (34)^d^
OEC-07-G3	Endometrioid	Grade 3	3C1	Carbo	Alive and well (24)
OEC-24-G3	Endometrioid	Grade 3	2	No	Alive and well (29)
OEC-09-SC/N	Serous		1 A	Carbo	Alive and well (38)
OEC-10-SC	Serous		1B	Carbo + PXL	Alive and well (22)
OEC-04-CC/E	Clear cell		1B	Carbo + PXL	Alive and well (32)
OEC-14-CC^a^	Clear cell, region 1		1 A	Carbo	Alive and well (15)
OEC-13-CC^a^	Clear cell, region 2		1 A	Carbo	Alive and well (15)
OEC-15-CC^a^	Clear cell, region 3		1 A	Carbo	Alive and well (15)
OEC-16-CC^a^	Clear cell, region 4		1 A	Carbo	Alive and well (15)
OEC-17-CC^a^	Clear cell, region 5		1 A	Carbo	Alive and well (15)
OEC-19-CS	Carcinosarcoma		1B	Carbo	Relapsed (16)^e^

*G1* grade 1, *G2* grade 2, *G3* grade 3, *SC* serous carcinoma, *CC* clear cell, *CS* carcinosarcoma, *E* endometrioid, *N* normal, *PXL* Paclitaxel, *Carbo*: Carboplatin.

^a^Organoids derived from different regions of the same patient tumor.

^b^Relapse vagina 9 months after primary treatment, radiation with complete response.

^c^Last follow-up data missing

^d^Relapse vagina 18 months after primary treatment, patient is awaiting response evaluation from radiation therapy.

^e^Relapse with lung metastasis 3 months after primary treatment. Progression on Tamoxifen and Doxorubicin given subsequently. Gemcitabine plus Docetaxel will be administered.

### Establishment of organoid-based patient-derived xenografts (O-PDX)

NOD.Cg-Prkdc scid IL2rg tm1WjI/SzJ (NSG) mice were purchased (Scanbur) and housed in individually ventilated cages. Mice were fed a low-autofluorescence imaging diet (D10001, Research Diets Inc., New Brunswick, NJ, USA) and had *ad libitum* access to food and water. Organoids (O-early) were passaged and cultured for 3–7 days before implantation. The organoids were removed from Matrigel and gently dissociated by pipetting up and down, followed by re-suspension in Matrigel. A volume of 30–50 μl organoid:Matrigel suspension (1:1) was orthotopically implanted in the left uterine horn as previously described^14,30^. The tumor growth was monitored by abdominal palpation and in vivo small animal imaging, using near-infrared fluorescent (NIRF) imaging or magnetic resonance imaging (MRI). Mice were sacrificed by cervical dislocation following clinical symptoms of disease (lethargy, abdominal enlargement, clearly palpable uterine tumor, or weight loss of ≥10%). All animal experiments were conducted according to institutional guidelines, and ethical approval was granted from the Norwegian Food Safety Authority (FOTS IDs 6710,12825, and 20194). All animal experiments are reported in accordance to ARRIVE guidelines. O-PDX models are indicated with the nomenclature OPDX-number-type/grade.

### Imaging of O-PDX models

NIRF imaging was performed using a fluorochrome-conjugated antibody targeting the epithelial cell adherence molecule EpCAM (EpCAM-AF680)^30^. Briefly, mice were injected with 60 µg EpCAM-AF680 in the tail vein 24 hours before imaging. Images were acquired using an Optix MX3 Time-Domain Optical Imager (ART Inc., Saint-Laurent, QC, Canada) and analyzed using the Optix OptiView software (version 2.02; ART Inc., Saint-Laurent, QC, Canada). Region of interests (ROI) was manually drawn around tumors before removal of background signal and measurement of the total fluorescent signal.

MR images were acquired on a 7 Tesla MRI (Pharmascan, Bruker) using a mouse body quadrature volume resonator in a single-coil configuration. Mice were anesthetized by sevoflurane mixed in oxygen and monitored for breathing and temperature throughout the scan. T2 sequences were acquired coronally and encompassed the whole tumor (TE/TR 25/2500 ms, 5 averages, matrix 160 × 160, field of view (FOV) 32 × 32 mm, slice thickness 1 mm, resolution 0.2 × 0.2 mm). ROIs were manually drawn on each slice to calculate total tumor volume.

### Re-derivation of tumor tissue and xenograft-derived organoids

O-PDX derived organoids were established both from the primary uterine tumor and from macroscopically identified metastases of first-generation (F1) mice. Second generation (F2) O-PDX models were generated either by direct reimplantation of minced tumor tissue from F1 mice or by implantation of F1-derived organoids after 2–4 in vitro passages.

### Morphological evaluation and immunohistochemistry

Organoid:Matrigel droplets were incubated in a cell recovery solution (VWR) for 1 h to remove the Matrigel. Organoids were formalin fixed for 10 min, washed once in PBS, and resuspended in 60 µl bovine plasma (Sigma Aldrich). For coagulation, 30 µl bovine Thrombin (Merck) was added. The pellet was paraffin embedded and 4 µm sections were cut. Hematoxylin and Eosin (H&E) stained slides were examined for histological evaluation by a pathologist. Biomarker expression was assessed by a standard immunohistochemistry protocol (IHC)^31^. Paraffin sections of organoids, corresponding patient biopsies and mouse tissue were incubated with anti-estrogen receptor (ER) (Dako), anti-progesterone receptor (PR) (Dako), anti-p53 (Dako), anti-epithelial cell adhesion molecule (EpCAM) (Cell signaling), anti-L1 cell adhesion molecule (L1CAM) (BioLegend, SIG-3911), anti-phosphatase and tensin homolog (PTEN) (Cell Signaling), anti-AT-rich interactive domain-containing protein 1 A (ARID1A) (Abcam), anti-mutS homolog 6 (MSH6) (Leica), and anti-mutS homolog 2 (MSH2) (Leica), anti-PMS1 homolog 2 (PMS2) (Leica), anti-mutL homolog 1 (MLH1) (Leica), and anti-Ki67 (Abcam). Sections were incubated for 30 min with anti-rabbit or anti-mouse secondary HRP-conjugated antibody (Dako), before the addition of DAB-chromogen (Dako) and hematoxylin. Staining conditions are listed in Supplementary Table 1. P53 protein levels were scored as normal or abnormal expression, as described previously^32^. ARID1A and PTEN were scored as intact or loss of expression. All other markers were categorized based on a percentage of positive cells, i.e., negative expression: <10% cells with positive staining, heterogenous expression: 10–60% cells with positive staining, or positive expression: >60% of cells with positive staining.

### Imaging mass cytometry (IMC)

Paraffin sections from organoids at early passage were deparaffinized and rehydrated prior to staining with a cocktail of 26 metal-conjugated antibodies (Supplementary Table 2). Briefly, antigens were retrieved in Tris/EDTA-buffer pH9 (Dako) at 96 °C for 30 min. Slides were blocked in 3% BSA in PBS for 45 minutes, followed by incubation with antibody cocktail overnight at 4 °C. Slides were washed once in 0.2% Triton X-100 in PBS and twice in PBS, before incubation with Intercalator-Ir (Fluidigm) (1:300) for 30 min. Slides were washed and air-dried before image acquisition by IMC using CyTOF Hyperion (Fluidigm). Image acquisition was performed according to the manufacturer’s instruction using a laser frequency of 200 Hz.

Images were visualized in the MCD Viewer v.1.0.5 (Fluidigm). The cell boundaries were segmented using Illastik v.1.3.3 and CellProfiler v.3.1.9. Pixels were classified in Illastik using a combination of nuclear and membrane staining to generate probability maps. Images were subsequently segmented into single cells in CellProfiler. TIFF images with single-cell segmentation masks and mean pixel expression values were extracted and used to generate tSNE plots in HistoCAT v.8.4.0. Single-cell expression values were normalized between 0 and 1 in R for visualization of marker heat on tSNE plot. Single cells were clustered based on selected known EC tumor markers using the unsupervised clustering algorithm PhenoGraph^33^ and 50 nearest neighbors. Markers chosen for clustering include Vimentin, EpCAM, Pan-cytokeratin, ER, PR, E-cadherin, β-catenin, Ki-67, Collagen type I, pERK1/2, and pS6. Phenograph identified 19 clusters, which were merged based on similar or biologically relevant phenotypes into seven distinct cell phenotypes. To correlate IHC and IMC markers, single cells were manually gated on biaxial scatterplots of mutually exclusive markers in HistoCAT. Percent positive cells found by IMC were correlated to percent positive cells determined by IHC using Spearman correlation in Graphpad Prism v.8.0.1.

### In vitro and in vivo drug treatment

Organoids at early passage were dissociated into small cell aggregates and resuspended in expansion medium with 5% GFR Matrigel. Depending on the organoid line, 10,000–20,000 cells were seeded in 96-well opaque-walled white plates precoated with 40 µl of GFR Matrigel and left to grow for 48 h. Cells were treated for 48 h with different concentrations of Carboplatin (100/200 µM) (Merck), Paclitaxel (10-200 nM) (Merck), Carboplatin-Paclitaxel (100/200 µM Carboplatin + 200 nM Paclitaxel), or DMSO. Selected doses are in line with clinically relevant doses, with maximum serum concentration of Carboplatin and Paclitaxel after intravenous infusion at 135 and 4.27 µM, respectively^34^. Cell viability was measured using CellTiter-Glo^®^ 3D Cell Viability Assay (Promega, G9682). The luminescence signal was normalized to the signal of untreated cells.

For in vivo treatment, 24 female NSG mice (8–10 weeks old) were orthotopically implanted with 2 ×10^6^ cells (OEC-07-G3, p14) per mice. The tumor growth was monitored weekly by T2-weighted MRI, and mice were randomized into two groups when tumor volume reached >145 mm^3^. Twice a week, the treatment and control groups were administered intraperitoneally with either 15 mg/kg Carboplatin plus 12 mg/kg Paclitaxel^35^ (*n* = 11) or saline (*n* = 13), respectively. Mice were sacrificed when reaching the humane endpoint or after 5 weeks of treatment. Response to Carboplatin-Paclitaxel combination therapy was evaluated by tumor volume (measured by T2-weighted MRI) and by postmortem observations of tumor size and metastatic lesions.

### Whole exome sequencing (WES)

DNA was extracted from snap-frozen patient tissue, organoids, and snap-frozen O-PDX biopsies using AllPrep DNA/RNA mini kit (QIAGEN) according to the manufacturer’s protocol. Tumor purity was >95% for organoids, >80% for O-PDX biopsies, and >75% for patient tissue, except for EC-08 (60%), EC-04 (50%) and EC-05 (60%).

Libraries were prepared using KAPA Hyper Prep (100 ng input) and captured using SeqCap EZ MedExome from Roche. A subset of samples was prepared using Twist Human Core Exome (50 ng input) with enzymatic fragmentation and RefSeq optional panel included. The sequencing was performed by Illumina Hiseq 4000 (chemistry MedExome: 150 × 2, Twist: 75 × 2). The raw reads quality was assessed using FASTQC. The alignment was performed using bwa-mem 0.7.17 against human Genome GRCh38, the aligned bam files were processed using samtools 1.4.1. Duplication was removed using Picard v.2.17.0.

Ontarget capture was calculated using bed tool intersect (version 2.27.1) where the coordinates overlap was calculated using exome MedExome hg38 bed files provided by Roche. The Depth and coverage analysis were performed using GATK version 3.8, which were further parsed to be used for variant calling using samtools mpileup module and Varscan-2.4.3. The cut-off values for normal samples were 10 reads, and 14 for tumor samples with a minimum variant frequency of 0.02. High confidence somatic variants selected by Varscan-2.4.3 were annotated using Annovar (version 2018-04-16) with hg38 reference files. Annotated variants are represented in the results section. The R/Bioconductor package Maftools was used to display oncoplots, mutation burden, and nucleotide substitution frequency. We used FACET to estimate total copy-number alterations, from joint segmentation of total- and allele-specific read counts, and integer copy-number calls corrected for tumor purity, ploidy, and clonal heterogeneity^36^.

### Sanger sequencing

Sanger sequencing (3730*xl* DNA Analyzer 3730xl, Applied Biosystems) was performed using PCR products of *POLE* exon 9, 11, 13, and 14 (Supplementary Table 3). Sequences were analyzed using DNA Sequencing Software (Chromas, v.2.6.6) and by manual inspection of chromatograms. A mutation was considered pathogenic if identical to one of the following alterations: p.P286R, p.A456P, p.V411L, p.F367V, p.S297F, p.F367S, and p.P436R^37^.

### RNA sequencing

RNA was extracted from organoids at early passage using AllPrep DNA/RNA mini kit (QIAGEN) according to the manufacturer’s protocol. cDNA libraries were prepared using Illumina TruSeq Stranded Total RNA GOLD (350 ng RNA input), and sequencing performed by Illumina HiSeq 4000 (paired end, 75 bp). Raw RNA-Seq reads were aligned to human genome GRCh38 using hisat 2.0.5 with Gencode v26 transcriptome reference. Aligned files were processed using Samtools. Reads aligned in the coding regions of the genome were counted using Feature Counts. Read counts were normalized using DESeq2 and transcripts with a sample mean log2 value >5 were selected for downstream analysis.

The 200 transcripts with the highest variance between the samples were subjected to unsupervised hierarchical cluster analysis in the R/Bioconductor programming environment. Significant analysis of microarrays (SAMs) was performed between dendrogram Cluster 1 and Cluster 2 in J-Express software (Molmine, Bergen, Norway). A fold change of 1.5 and FDR < 0.1% were used as a cut-off. The list of differentially expressed genes was used to develop a gene signature in an independent set of 256 EC patients, with gene-expression data available from a microarray analysis performed as described previously^38^. Four of the probes for the significantly differentially expressed genes were not present in the microarray dataset and were omitted from the gene signature. The gene signature score was calculated by subtracting the average expression levels of upregulated genes from the average expression levels of downregulated genes across the samples. Dichotomization was performed using the mean gene signature score.

### Validation cohorts

The L1000 transcriptional profiling was performed as previously described^39^. Briefly, RNA was added to oligo dT-coated 384-well Turbocapture plates. Reverse transcription mix with Moloney Murine Leukemia Virus Reverse Transcriptase was added before washing and addition of upstream and downstream probes for each gene. Probes were designed with a universal primer site, a gene-specific primer, and a unique barcode sequence. The PCR product was hybridized to Luminex microbeads with complementary barcodes on each bead. After overnight incubation, beads were stained with streptavidin–phycoerythrin followed by detection with Luminex FlexMap 3D scanners. The 978 directly measured landmark genes were then extrapolated using an algorithm to generate a transcription profile of 12,328 genes. Replicate-collapsed *z*-scores (level 5 data) were used for downstream analyses. The Uterine Corpus Carcinoma (TCGA, PanCancer Atlas, *n* = 527) transcriptomic dataset (RNA-seq) was downloaded from cBioPortal (https://www.cbioportal.org/datasets).

### Statistics and reproducibility

Survival differences between groups were assessed by Kaplan–Meier using the Mantel-Cox (log-rank) test. Death due to endometrial cancer was defined as an event for analysis of disease-specific survival. Pearson’s *X*^2^ test was used to evaluate correlations between categorical variables. Spearman correlation analysis was performed to test the relationship between percent positive cells measured by IMC and IHC. Statistical difference between MRI tumor size was calculated individually for each treatment group/week using paired *t* tests. Above ten mice in each group were considered enough to evaluate the treatment effect. Normality was tested using Shapiro–Wilk test. Statistical significance was determined without correction for multiple comparisons, with alpha = 0.05. For in vitro treatment experiments, ≥2 independent experiments were considered enough to demonstrate inter-organoid heterogeneity in drug response. Analyses were carried out in SPSS-26 (IBM, New York) and GraphPad Prism v.8.0.1.

## Results

### EC organoids capture disease heterogeneity

**Fig. 1:**
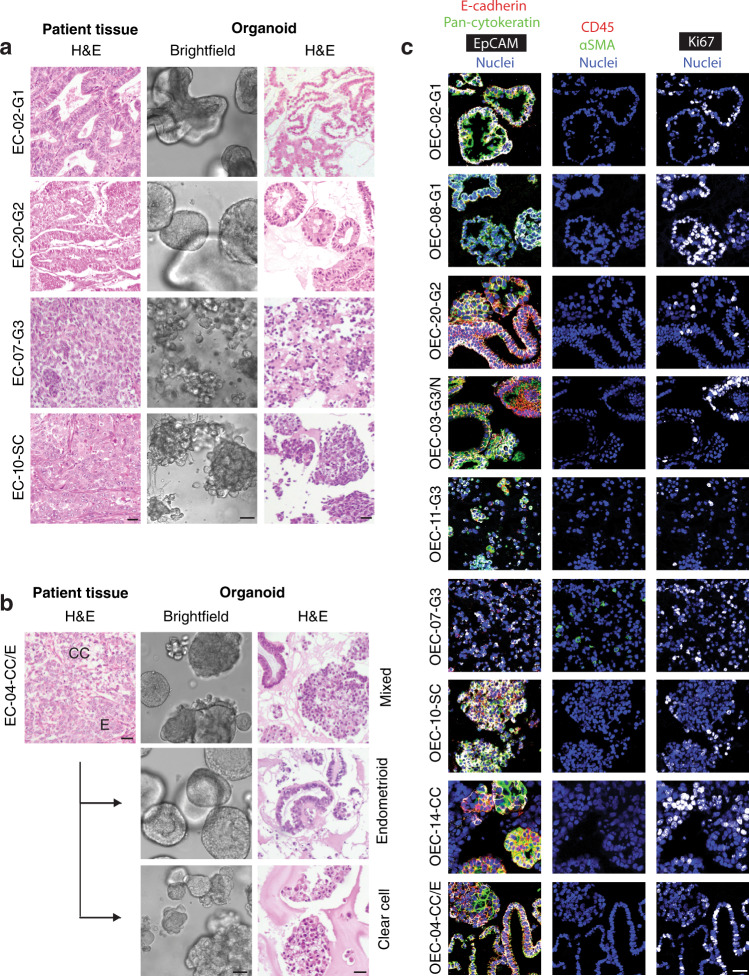
Tumor organoid cultures share histopathological features with corresponding patient tissue. **a** Comparative H&E images of selected patient tissues with brightfield and H&E image of corresponding organoid cultures. **b** EC-04-CC/E patient tumor with both clear cell and endometrioid components was used to generate mixed histology organoids, shown in brightfield and H&E in the upper panel. The OEC-04-CC/E culture was subcloned into endometrioid organoids (middle panel) and clear cell organoids (lower panel) to preserve both tumor components in culture. **c** Multiplexed immunohistochemistry images of organoids stained with markers for epithelial cells (E-cadherin: red, pan-cytokeratin: green and EpCAM: white), leukocytes (CD45: red), fibroblasts (αSMA: green), and cell proliferation (Ki67: white). The heterogeneous expression of epithelial markers is seen within and between all cultures, while the cultures were negative for non-epithelial markers, indicating pure cancer cultures. Scale bars = 20 µm.

Endometrial cancer organoid specific medium was developed from normal endometrial expansion medium^40^ by removing N2-supplement. Withdrawal experiments indicated that R-Spondin, A83-01, p38 inhibitor, and ROCK inhibitor were required for optimal growth (Supplementary Figure 1a). The final medium is described in the material and methods section and was further used to successfully establish and long-term culture organoids from all types and grades of endometrial cancer (Table 1, Supplementary Figure 1b). The efficacy of establishment, defined as organoid expansion for more than four passages, was 22% when including all available tumor samples (Supplementary Data 1). One donor was sampled at different tumor regions, and organoids were successfully established from all five tumor biopsies (Table 1). The morphological organization of organoid models resembled the histology of the donor patient tissue, as illustrated for selected organoid lines in Fig. 1a. Typical glandular structures were seen in organoids from low-grade (grade 1–2) endometrioid endometrial cancers while more solid organoid growth with endometrioid features was present in endometrioid high-grade subtypes. Organoids from clear cell and serous subtypes showed high proliferation and less glandular differentiation with histology consistent with the patient donor tissue. Mixed histology was seen in EC-04-CC/E containing groups of cells with clear cell morphology mixed with malignant glands of endometrioid subtype.

Organoid cultures were closely monitored to ensure consistent morphology over time. In three cultures, including one endometrioid grade 3 and two clear cell non-endometrioid, cells with less malignant morphology were observed, also matching the histological findings in the donor tumor (Supplementary Figure 2). In organoid cultures derived from mixed histology tumors, less malignant organoids were favored over time in culture, indicating a more efficient regeneration of well-differentiated organoids. To preserve both morphologies, early stage mixed cultures were split by manually selecting organoids based on morphology. The subcloned cultures efficiently expanded and maintained subtype-specific morphology (as exemplified in Fig. 1b).

Imaging mass cytometry of the organoid cultures revealed positive and heterogeneous expression of epithelial markers (E-cadherin, pan-cytokeratin, and EpCAM) and negative expression of CD45 and αSMA, reflecting pure epithelial organoid cultures (Fig. 1c). Variations in proliferation rates between the cultures were indicated by differences in Ki67 staining, also in line with observations of growth rate in culture and IHC Ki67 staining (Fig. 1c and Supplementary Figure 1c, d).

### Protein marker profiles in organoids match EC subtype-specific expression patterns

**Fig. 2:**
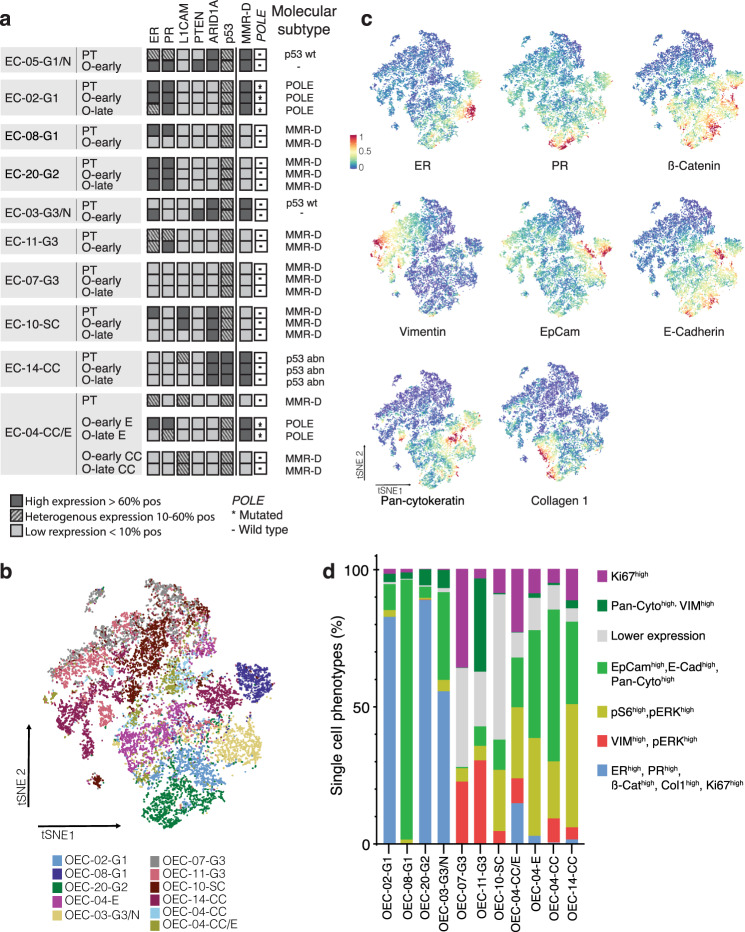
Organoid models mirror biomarker status and molecular subgroup of donor tissue. **a** Expression levels in paired patient tissue (PT) and early (and late-) passaged organoids (O-early and O-late). Samples were classified according to the ProMiSE classifier: DNA mismatch repair deficiency (MMR-D) (loss of one or more MMR proteins), hotspot mutations in the exonuclease domain of DNA polymerase epsilon (*POLE)* (detected by Sanger sequencing) and p53 wt or mutated based on aberrant immunohistochemical staining patterns. Samples were not classified if they had low tumor purity. AT-rich interactive domain-containing protein 1 A (ARID1A) and phosphatase and tensin homolog (PTEN) were scored as homogenous positive (dark gray) or loss of (light gray) expression. All other markers were scored according to percentage positive expression as indicated in the figure. Estrogen receptor (ER), progesterone receptor (ER), epithelial cell adhesion molecule (EpCAM), L1 cell adhesion molecule (L1CAM). **b** Map using *t*-distributed stochastic neighbor embedding (*t*SNE) of single cells from CyTOF Hyperion images (26-markers) of EC organoid samples colored by cluster identifier. **c** *t*SNE plots showing Vimentin, EpCAM, E-cadherin, ER, PR, and β-catenin expression from all samples using a 0 to 1 normalization. **d** Stacked bar plot of single-cell phenotype densities in each organoid culture. Cell phenotypes are defined by combined PhenoGraph clusters.

The patient tissue and corresponding organoid models were profiled for protein expression of relevant endometrial cancer markers: ER, PR, L1CAM, PTEN, ARID1A, and assigned to molecular subgroups according to the TCGA-like classification by evaluating IHC staining of P53 and MMR proteins (MSH6, MSH2, PMS2, and MLH1), and by Sanger sequencing of *POLE*^41^ (Fig. 2a). Representative images of expression patterns are shown in Supplementary Figure 3. With few exceptions, the expression patterns of the organoids matched the typical expression for the molecular subtype and reflected the expression patterns of the paired primary tumor. The expression of hormone receptors was lower compared to the corresponding patient tumor in both OEC-08-G1 and OEC-10-SC organoids. The expression patterns of ER, PR, ARID1A, and L1CAM for OEC-04-CC/E were discordant, likely due to the observed organoid sub-clonality.

Subtype-specific organoid phenotypes were further confirmed when single-cell data from imaging mass cytometry was analyzed in detail (Fig. 2b and Supplementary Figure 4a). tSNE plots of single-cell segmentation masks of organoids identified two main groups that reflected different histological subtypes (Fig. 2b). The lower grouping comprised endometrioid G1-2 subtype organoids with distinct subgroups for each culture, and the upper grouping contained endometrioid G3 and non-endometrioid subtypes with less distinct subgroups. Interestingly, the subcloned OEC-04-CC/E culture was distributed between the two main groups, with the endometrioid component closer to the low-grade organoids. Single-cell expression values for specific markers are shown in Fig. 2c, indicating overlapping expression of hormone receptors and ß-catenin in low-grade tumors, and a corresponding loss in higher-grade tumors. To further explore single-cell phenotypes, masked single cells were clustered based on the expression of the tumor markers Vimentin, EpCAM, Pan-cytokeratin, ER, PR, E-cadherin, β-catenin, Ki-67, Collagen type I, pERK1/2, and pS6. Nineteen cell clusters were identified and further grouped into seven distinct cellular phenotypes based on expression similarities. Interestingly, samples had unique cellular compositions (Fig. 2d). Distinct cellular phenotype patterns were associated with different grades, including a higher fraction of Ki67^high^, pERK^high^, and pS6^high^ in grade three EECs, serous, and clear cell organoids. ER, PR, EpCAM, and Ki67 expression data were available from both IHC and IMC and showed high concordance in most organoids (Supplementary Figure 4b).

### EC organoids engraft to generate orthotopic O-PDX models

**Fig. 3:**
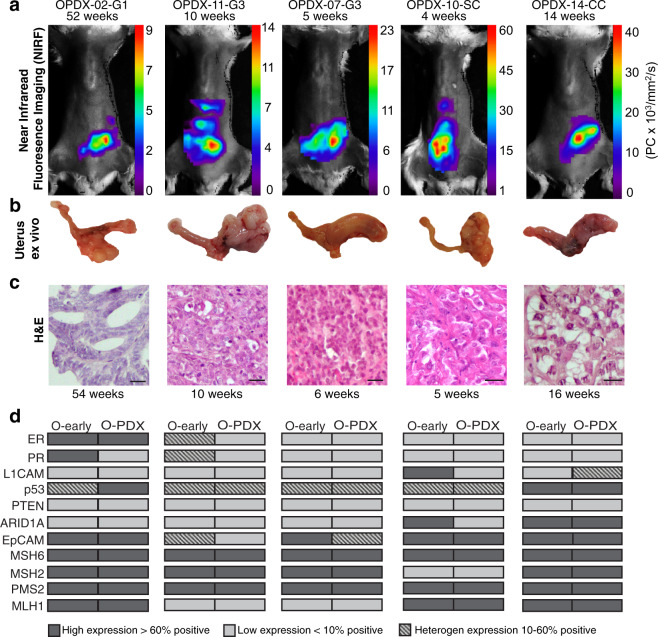
Organoids are successfully engrafted orthotopically in NSG mice. **a** NIRF imaging of orthotopically implanted organoids in NSG mice illustrating the presence of tumor in lower abdomen 4–52 weeks after implantation. **b** Ex vivo images depicting tumor burden in the uterus. **c** H&E section of xenografts showing histological subtype characteristics. The number of weeks from implantation to sacrifice are indicated. **d** Panel of immunohistochemical marker expression in the organoid implant (O-early) and corresponding organoid-based patient-derived xenograft (O-PDX). Protein abbreviations are as follows, estrogen receptor (ER); progesterone receptor (PR); L1 cell adhesion molecule (L1CAM); phosphatase and tensin homolog (PTEN); AT-rich interactive domain-containing protein 1A (ARID1A); epithelial cell adhesion molecule (EpCAM); mutS homolog 6 (MSH6); mutS homolog 2 (MSH2); PMS1 homolog 2 (PMS2); MLH1 mutL homolog 1 (MLH1). Markers were scored as described in Fig. 2. Scale bars = 20 µm.

Organoids representing the spectrum of histologic types of EC were selected for orthotopic implantation. Eight of ten organoid lines, including endometrioid grade 1 (*n* = 1), grade 2 (*n* = 1), grade 3 (*n* = 3), and non-endometrioid serous (*n* = 1) and clear cell (*n* = 2), formed uterine tumors in mice, yielding an engraftment rate of 80% (Table 2). Disease development in O-PDX models was monitored by in vivo EpCAM-AF680 NIRF^30^ (Fig. 3a) or MRI^14^. Strong fluorescent signals were detected in the lower abdomen of the O-PDX mice 4–52 weeks after implantation, suggesting the presence of uterine tumors. In general, tumor development was slower for lower-grade tumors (mean 69 weeks from implantation to sacrifice for grade 1 and 2) and more rapid for higher-grade and non-endometrioid tumors (mean 18 weeks) (Table 2). Post-mortem examinations, including histologic evaluation of xenograft tissue confirmed the presence of endometrial tumor tissue (Fig. 3b), which reflected the histologic subtype (Fig. 3c) of the donor tissue. Metastatic lesions were detected in several mice, including metastases to pelvic or renal lymph nodes, ovary, kidney, pancreas, diaphragm, and/or liver (Supplementary Figure 5a). Organoids were successfully established from the xenograft biopsies and validated to reflect the histology of their donor tissue (Supplementary Figure 5b).

**Table 2:** Established orthotopic O-PDX models.

	Weeks^a^	
O-PDX ID	F1 (F2)	Metastasis
OPDX-02-G1	54	No
OPDX-20-G2	29	Yes
OPDX-07-G3	6 (5)	Yes
OPDX-11-G3	10	Yes
OPDX-24-G3	37	No
OPDX-10-SC	5 (7)	Yes
OPDX-14-CC	16	No
OPDX-04-CC/E	37	Yes

^a^time from organoid implantation to clinical endpoint.

The biomarker status of the xenografts was similar as in the organoids (Fig. 3d), with few exceptions. Specifically, hormone receptors were lost and L1CAM expression altered in some xenografts. Sequential re-derivation of tumor tissue to F2 generation mice did not alter disease-course or histologic features. Long-term cultured organoids (10 months) were confirmed to form tumor in mice (*n* = 3), with uterine tumors and macroscopically visible metastatic lesions (renal lymph node, diaphragm, and pancreas) (Supplementary Figure 5c).

### Organoid-based EC models show individual response to conventional therapy

**Fig. 4:**
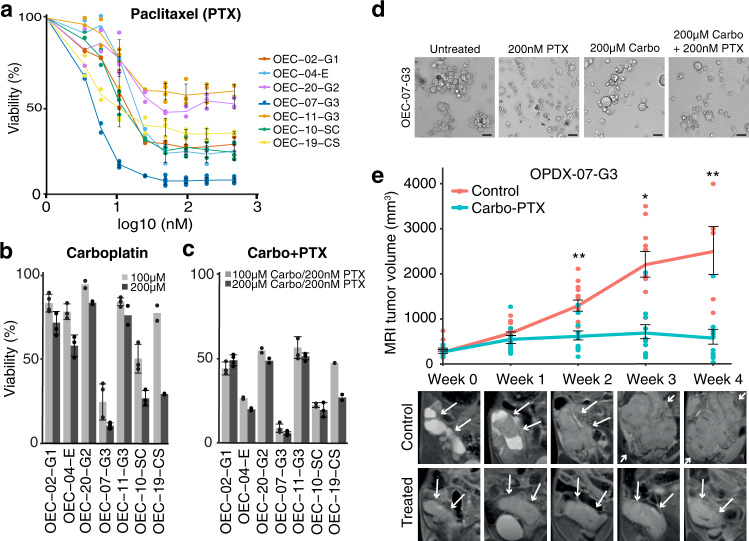
Drug response in organoids can be reproduced in the corresponding O-PDX model. **a–c** Seven organoid lineages were treated with different concentrations of Carboplatin (Carbo) and/or Paclitaxel (PTX). Viability was measured with CellTiter-Glo 3D Assay after 48 hours. Standard deviation was calculated from *n* ≥ 3 independent experiments. **d** Representative brightfield images depicting morphology of OEC-07-G3 untreated or after 48 h treatment with Carboplatin and/or Paclitaxel. **e** Growth of OPDX-07-G3 xenografts treated with 15 mg/kg Carboplatin and 12 mg/kg Paclitaxel (*n* = 11 mice) or saline (*n* = 13 mice) twice a week for five weeks (mean $\pm \,$SEM). Treatment started when tumors reached >145 mm^3^ on T2-weighted MR images. Paired sample *t* test, *p < 0.05, **p < 0.001. Representative longitudinal T2-weighted MR images of xenografts showing uterine tumor size in treated vs. untreated mice. Scale bars = 20 µm.

Individual organoids showed differential drug sensitivity to Paclitaxel, Carboplatin, and Carboplatin-Paclitaxel, and most lines showed a poor response (Fig. 4a–d). OEC-07-G3 was highly sensitive to Carboplatin-Paclitaxel with only 5.4% viability measured after combination treatment with Carboplatin (200 µM)-Paclitaxel (200 nM). To validate a similar response in vivo, NSG mice were orthotopically implanted with OEC-07-G3 and treated with Carboplatin-Paclitaxel (*n* = 11 mice) or saline (*n* = 13 mice). After 5 weeks of treatment, an average increase of 1941 mm^3^ and 156 mm^3^ in tumor volume was measured on T2-weighted MRI in control and treated mice, respectively (*p* = 0.001) (Fig. 4e). At necropsy, macroscopically visible metastatic lesions were observed in 12/13 of the untreated mice, including organs, such as the pancreas, liver, renal lymph nodes, diaphragm, or mesentery. In the treatment group, metastatic lesions were observed in 2/11 mice only (Table 3). Combined, these results demonstrate similar drug responses between our in vitro and in vivo model systems.

**Table 3:** Presence of metastases in O-PDX-07-G3 mice treated with Carboplatin-Paclitaxel versus saline control.

	O-PDX ID	Metastastatic site
**Treatment group**	O-PDX-07-G3-B1	Pancreas
	O-PDX-07-G3-B2	Liver
	O-PDX-07-G3-B3	No metastases
	O-PDX-07-G3-B4	No metastases
	O-PDX-07-G3-B5	No metastases
	O-PDX-07-G3-B6	No metastases
	O-PDX-07-G3-B7	No metastases
	O-PDX-07-G3-B8	No metastases
	O-PDX-07-G3-B9	No metastases
	O-PDX-07-G3-B10	No metastases
	O-PDX-07-G3-B11	No metastases
**Control group**	O-PDX-07-G3-A1	Liver, pancreas
	O-PDX-07-G3-A2	Pancreas, mesentery
	O-PDX-07-G3-A3	Pancreas, liver
	O-PDX-07-G3-A4	Pancreas, liver
	O-PDX-07-G3-A5	Pancreas, liver
	O-PDX-07-G3-A6	Pancreas, liver, renal ln.
	O-PDX-07-G3-A7	Pancreas, liver, renal ln.
	O-PDX-07-G3-A8	Pancreas, renal ln., diaphragm
	O-PDX-07-G3-A9	Pancreas
	O-PDX-07-G3-A10	Pancreas, liver, diaphragm
	O-PDX-07-G3-A11	Pancreas, liver, diaphragm
	O-PDX-07-G3-A12	Pancreas, diaphragm
	O-PDX-07-G3-A13	No metastases

Renal ln.: lymph nodes nearby the renal blood vessels, dorsal to the ipsilateral kidney, and caudal to the adrenal gland.

### EC organoids and O-PDXs retain the mutation profile of the corresponding primary tumor

**Fig. 5:**
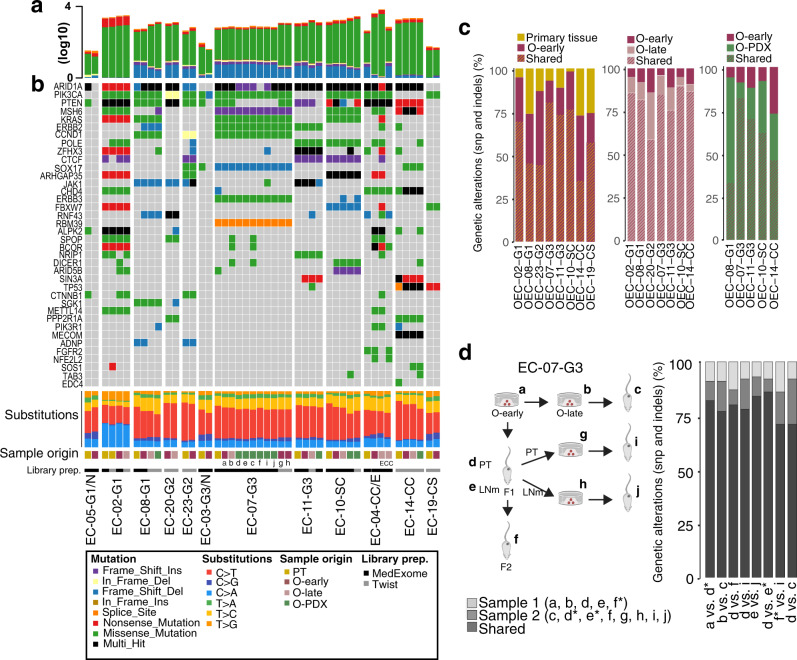
Organoids and O-PDX models have a shared genomic landscape with corresponding patient tumors. **a** Histogram denotes the total mutation burden in each sample (log10). **b** Oncoplot of somatic EC driver alterations and nucleotide substitution frequency in paired patient tissue (PT), organoid models (O-early and O-late), and organoid-based patient-derived xenograft (O-PDX) models. Sample annotation is indicated in the lower panel. Samples were prepared using MedExome or Twist capture systems as indicated in Figure. See method section for details and Supplementary Figure 6c for comparisons of the two methodologies. **c** Stacked bar charts showing overlap of nonsynonymous mutations in PT vs corresponding O-early, O-early vs O-late, and O-early vs O-PDX. **d** EC-07-G3 sample overview showing patient-derived (**a**, **b**) and xenograft-derived organoids (**g**, **h**), O-PDX models generated from early and late-passaged organoids (**c**, **d**, **e**), F2 generation O-PDX model generated by direct reimplantation of xenograft tissue (**f**) or by implanting xenograft-derived organoids (**i**, **j**). The stacked bar chart shows the overlap of nonsynonymous mutations between selected sample pairs from EC-07-G3.

The spectrum of somatic mutational aberrations in corresponding samples (*n* = 13 sample sets: patient tissue, early/late-passaged organoids, and O-PDXs) was explored by WES. This included one set with 11 different samples derived from EC-07-G3: organoids at an early and late stage, xenografts generated from early and late-stage organoids, mouse lymph node metastasis, xenograft-derived organoids from both uterine tumor and lymph node metastasis and the xenograft generated by orthotopically injecting these organoids (Fig. 5d; a–j). Mean depth of sequencing reads were 157, 183, 174, and 164 in patient tissue, O-early, O-late, and O-PDXs, respectively. In general, the total mutational burden was similar between the corresponding samples (Fig. 5a). One exception was the early stage, nonsplit organoid OEC-04-CC/E, which had a substantially higher number of mutations than its primary tumor counterpart. Sequencing the late-stage pure endometrioid component revealed a similar high mutation burden as the early stage, nonsplit culture, while the clear cell component showed a much lower mutation frequency, similar to the donor.

A panel of major EC driver genes was defined based on previous work^42^ and explored for alterations. *ARID1A*, *PIK3CA*, and *PTEN* were most frequently mutated in our samples, followed by mutations in *MSH6*, *KRAS*, *ERBB2*, and *CCND1* (Fig. 5b). Other driver mutations were spread among the organoid/O-PDXs, reflecting heterogeneity between the lines. Mutational profiles were highly concordant between paired samples (primary tumors, organoids, and O-PDXs). This was clearly demonstrated with the 11 samples from EC-07-G3, where the mutational profile was stable even after the implantation of late-passaged organoids, reimplantation of O-PDX-derived organoids, and after re-derivation of xenograft tissue. The OEC-03-G3/N and OEC-05-G1/N cultures where normal organoids dominated harbored one mutation in *PIK3CA* and no driver mutations, respectively. As expected, this is in dis-concordance with the paired primary tumor sample and reflects the expansion of nonmalignant cells. Nucleotide substitution frequency was also concordant between samples. We identified C > T substitutions as most frequent, followed by C > A and T > C substitutions. In patient EC-02-G1 however, a stronger component of C > A substitutions was identified (Fig. 5b).

The total number of nonsynonymous mutations between the primary tissue and O-early overlapped in the range of 35–80% (Fig. 5c). Importantly, the overlap between early and late cultures was >80% in most cases, suggesting that EC organoids are genetically stable over time in culture. Of the O-PDX samples, we identified on average 60% mutation concordance with the organoid implant, and >79% overlap between EC-07-G3 sample pairs (Fig. 5d). Copy-number profiles were similar between paired samples (primary tumors and corresponding organoids) (Supplementary Figure 6a), although some variations were seen after repeated implantation in mice for EC-07-G3 (Supplementary Figure 6b).

### RNA expression profile in organoids identifies prognostic gene signature

**Fig. 6:**
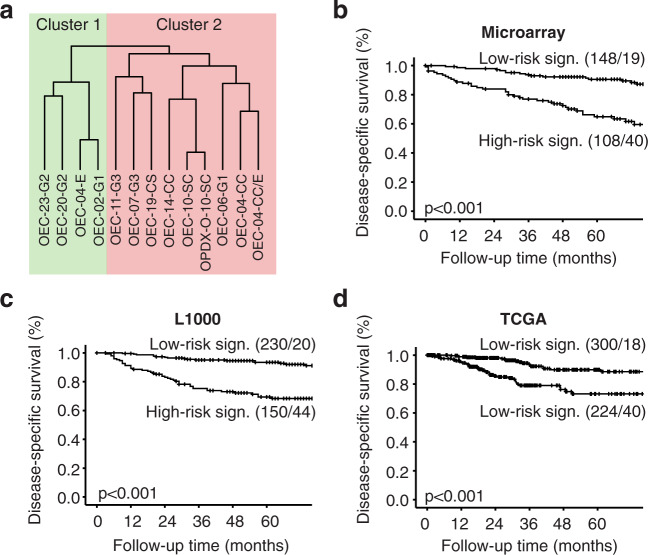
Organoid gene-expression signature predicts survival in endometrial cancer patients. **a** Dendrogram constructed by unsupervised hierarchical clustering of 13 organoid samples. OEC-04-E clustered together with OEC-02-G1 rather than its clear cell and mixed culture counterparts. RNA expression profile is similar between patient-derived and xenograft-derived organoids from EC-10-SC. **b** Disease-specific survival (DSS) according to gene signature score in a local EC microarray cohort (*n* = 256), **c** L1000 cohort (*n* = 380) and **d** the transcriptomic TCGA cohort (*n* = 524). Gene signature was dichotomized using mean signature score. Kaplan–Meier curves are presented by comparing two categories (number of patients investigated/number of patient deaths).

A subset of 13 organoid cultures was subjected to RNA sequencing. Unsupervised hierarchical clustering identified two distinct clusters, strongly linked to histological subtypes of the models (Fig. 6a). Cluster 1 consists of endometrioid grade 1–2 organoids and cluster 2 of endometrioid grade 3, non-endometrioid and one endometrioid grade 1 organoid. The donor patient of the latter presented with metastasis to the colon at primary surgery. SAM analysis identified 23 differentially expressed genes (FDR < 0.1%, fold change >1.5) between the two clusters. This gene list was used to develop a 19-gene signature in a microarray dataset with 256 EC patients with complete follow-up and clinical data (see Method section and Supplementary Table 4). The dichotomized gene signature score could significantly predict disease-specific survival (*p* < 0.001) and correlated with clinicopathological variables, including histological type and grade, lymph node metastasis, and myometrial infiltration in these patients (all *p* < 0.05; Fig. 6b and Supplementary Table 5). These findings were validated in two larger patient cohorts, including a local L1000 cohort (*n* = 380) and the TCGA RNA-seq cohort (*n* = 524) (Fig. 6c, d, Supplementary Table 6). This is highly encouraging considering the small number of organoid samples needed to identify prognostically valuable information and underlines the predictive potential of these models.

## Discussion

A major challenge in endometrial cancer research is the lack of robust models that reflect the molecular subtypes and course of disease of endometrial cancers. Organoids have shown great potential as models for other cancer types^43^, and the organoid system can be combined with other technologies, such as single-cell applications and live-cell imaging to facilitate both basic and translational research^44,45^. Few well-characterized EC organoids have previously been reported^28^. Here we describe the generation and comprehensive characterization of a collection of EC organoids that models both common and rare subtypes of EC tumors and show the successful generation of orthotopic mouse models based on these. We demonstrate that drug response observed in vitro is mimicked in vivo. A detailed characterization of mutational status compared to donor tissue provide evidence for genetic stability. Finally, translational data from the organoids provide molecular information with direct relevance for the prediction of patient outcome, further supporting the clinical relevance of these models.

EC organoids were successfully established after modifying the growth conditions reported for normal endometrial organoids^40^. Specifically, we removed N2-supplement and added the ROCK inhibitor, which allowed for long-term expansion and cryopreservation of the organoids. Cell aggregates from the primary tissue typically formed malignant glandular structures or more solid organoids with histological characteristics similar to their donor tissue. Known EC driver mutations detected in the patient tissue were also present in the organoids, both at early timepoints and after 20 passages, thus demonstrating their genetic stability. Importantly, immunohistochemical profiling of relevant biomarkers combined with genetic profiling show that our models mimic the molecular profile of both the histological and molecular subtypes of EC tumors. Imaging mass cytometry also revealed cellular heterogeneity in samples, with the presence of different cellular phenotypes with distinct expression patterns. Interestingly, the pattern of cellular phenotypes defined by IMC appears to associate with different histological types and grades. This finding should be further investigated in patient samples. The models harbored mutations in the most commonly mutated EC driver genes^42^, including alterations in *PIK3CA, ARID1A, PTEN, KRAS*, and *ERBB2*. Low-grade (grade 1–2) endometrioid subtypes were hormone receptor positive, which included two *POLE* ultramutated cases. Hormone receptor expression decreased in some lower-grade models. However, loss of hormone receptors is well known in endometrial cancer^38,46,47^ and whether this loss is due to medium conditions or evolution of the cancer cells should be further studied. High-grade and non-endometrioid subtypes had increased expression of L1CAM, p53 alterations, *FBXW7* mutations, and loss of hormone receptor expression. EC-11-SC lacked *TP53* mutation commonly found in serous cancers and harbored mutations in several genes more commonly mutated in endometrioid subtypes. This highlights the added benefit of molecular profiling in the diagnosis of ECs and underlines that future clinical trials for EC should be based on molecular findings rather than histopathological diagnosis. Some discrepancies were observed, for example, the additional *TP53* mutations in both EC-14-CC and EC-11-G3 organoids. Sample purity, as well as tumor heterogeneity in donor samples may affect results; however, some evolution of the mutational landscape is also expected. Analysis of single base substitutions revealed that C > T alterations were most frequent across the samples, except for EC-02-G1 with a stronger component of C > A substitutions. Frequent C > T base substitutions is often associated with high age and is common for EC patients, whereas frequent C > A substitutions associates with smoking^48,49^. According to the patient record, patient EC-02-G1 was a former smoker indicating that this is a likely cause of the base substitution signature found in this patient.

Subtype-specific expression patterns were further confirmed by unsupervised clustering of RNA-seq data and by protein expression data; both methods distinguished the low-risk endometrioid from higher risk endometrioid and non-endometrioid subtypes. Multiregion sequencing studies have reported sample-specific mutations for several cancers^50–52^, reflecting tumor heterogeneity. Tumor heterogeneity or mixed histology are common features of endometrial cancer, complicating both diagnostic work and treatment. We observed several cultures with organoids of different histology, particularly a mixture of clear cell and endometrioid subtype. Biomarker- and mutation profiles clearly demonstrated histology-specific patterns, confirming intra-tumor heterogeneity that should be considered when developing targeted treatment strategies for these patients in the future^53^.

Combining easy drug screening using in vitro organoid models with in vivo O-PDX models to study systemic disease development and treatment effects could greatly advance the field of endometrial cancer drug testing. Our orthotopic O-PDX models represent the main subtypes of EC and mimic disease progression in patients, e.g., a short disease course was associated with high-grade subtypes. Vaginal bleeding is the most common presenting symptom of endometrial cancer in patients^54^ and was observed in several of the O-PDX models, which is less often reported in cell line-based endometrial cancer xenografts. Overall, O-PDX models had metastatic lesions in sites that are commonly seen in endometrial cancer patients, including lymph nodes and ovaries. Interestingly, mice from the O-PDX EC-07-G3 model mirrored the metastatic spread in the donor patient at the time of surgery with metastases to pelvic lymph nodes. Molecular profiling further demonstrated that immunohistochemical biomarker expression and mutational patterns are mostly unchanged after engraftment.

The relevance of our organoid models was further demonstrated by low sensitivity to conventional treatment, mirroring the clinical setting where 40–60% of EC patients are resistant to standard of care chemotherapy^55–57^. The EC-07-G3 organoid however was highly sensitive to Carboplatin-Paclitaxel treatment in vitro, a response which was mimicked in vivo in the corresponding O-PDX model. Interestingly, when reviewing the patient record, no recurrence has been reported 2 years postchemotherapy and surgery. A larger organoid-donor cohort combined with continued follow-up of donor patients will provide more detailed response data for future studies. We are the first to demonstrate that orthotopic O-PDX models for endometrial cancer can reproduce in vitro treatment response. Our findings thus support the use of organoids for in vitro drug screening with subsequent in vivo validation and testing of systemic drug effects of promising candidates in O-PDX models.

Unsupervised clustering of transcriptional data of 13 organoids identified a 19-gene signature that predicted disease-specific survival in independent EC patient cohorts. This strongly implies that subtype-specific expression profiles are sustained in vitro and are less affected by growth conditions in the culture. The signature for aggressive disease included low expression of genes related to hormone signaling, including the hormone receptors *ESR1* and *PGR*, whereas genes associated with increased proliferation and invasion, including *SORCS2*, *COL4A1*, and *S100A9*^58–60^ were highly expressed. To our knowledge, similar translational results have never been extracted from previously published models. We find it highly encouragingly that the relatively small sample size of EC organoids can provide prognostic information relevant for EC patient cohorts, clearly demonstrating the predictive potential of our models. This also implies that drug response signatures can be derived from these models and translated to the clinical setting. This should be investigated in future studies and may provide valuable information for selecting patients to appropriate treatment^61^.

There are still challenges to be resolved to improve cancer organoid cultures. This includes a low success rate for culture initiation of several cancer types, as well as the generation of models for rare subtypes^62,63^. We here demonstrate that our modified culture conditions enabled the establishment of all subtypes with an overall success rate of 22%, and the established EC organoids display relevant genetic- and protein expression patterns. However, due to the population-based nature of our study, the number of rare subtypes is low and derivation success rates for specific subtypes are therefore uncertain. Continued efforts should strive to include all spectra of EC molecular subtypes (e.g., endometrioid grade 3 p53mut). One challenge that limits the success of establishment is an overgrowth of normal organoids. Molecular alterations can provide clues to cancer organoid niche independencies and have been the suggested approach to favor the growth of cancer organoids. For instance, withdrawal of R-spondin and Nutlin-3 from *CTNNB1* and *TP53* mutated samples, respectively, are likely to select for cancer organoids^64,65^. Intra-tumor heterogeneity will however limit this approach, as selective media will favor subclones that harbor the specific alteration causing the niche independency. As an alternative, we suggest manually remove organoids with normal morphology at an early timepoint in culture to preserve the genetic heterogeneity of the organoids. This is mostly feasible for endometrial cancer cultures as normal and malignant organoids show distinct morphologies, although this will not necessarily be the case for every model. Developing methods for co-culturing of immune- and stromal cells with endometrial organoids would further expand the area of applications, e.g., by enabling testing of immunotherapy agents^66^.

In summary, we have established EC organoid models from all histological grades and subtypes that mimic key features and heterogeneity of endometrial tumors. Together with O-PDX models, this comprises a platform with broad applications in experimental and preclinical research, and combined with the growing biobank of EC organoids, enables a more personalized approach in preclinical drug studies. The striking prognostic potential of the identified gene signature is interesting and supports the strong clinical relevance for future drug testing studies in EC organoid models.

### Reporting Summary

Further information on research design is available in the Nature Research Reporting Summary linked to this article.

## Supplementary information

Supplementary Information

Supplementary Data 1

Supplementary Data 2

Description of Additional Supplementary Files

Reporting Summary

## Data Availability

Source data used to generate graphs and charts are included in Supplementary Data 2. Primer sequences used for POLE sequencing are included in the Supplementary Table 3. Transcriptomic datasets are available at ArrayExpress: RNA-seq dataset [E-MTAB-10664], Agilent Microarray dataset [E-MTAB-5017]^67^, L1000 dataset [E-MTAB-10668]. The TCGA dataset (PanCancer Atlas) can be accessed via cBioPortal (https://www.cbioportal.org/datasets). Patient consent does not allow for deposition of WES data in public/controlled access repositories. Interested researchers should contact C.K. (camilla.krakstad@uib.no) to inquire about access; requests for noncommercial academic use will be considered and require ethics review.

## References

[1] Siegel RL, Miller KD, Jemal A (2019). Cancer statistics, 2019. CA. Cancer J. Clin..

[2] Carcangiu, M. L. WHO classification of tumours of female reproductive organs, fourth edition. *Lyon: International Agency for Research on Cancer (I A R C)(UN)* (2014).

[3] Cancer Genome Atlas Research N (2013). Integrated genomic characterization of endometrial carcinoma. Nature.

[4] Marnitz, S. et al. A modern approach to endometrial carcinoma: will molecular classification improve precision medicine in the future? *Cancers (Basel)* **12**, 2577 (2020). 10.3390/cancers12092577 PMC7564776 32927671

[5] Concin, N. et al. ESGO/ESTRO/ESP Guidelines for the management of patients with endometrial carcinoma. *Int J Gynecol Cancer* **31**, 12–39 (2020). 10.1136/ijgc-2020-002230 33397713

[6] Salvesen HB, Haldorsen IS, Trovik J (2012). Markers for individualised therapy in endometrial carcinoma. Lancet. Oncol..

[7] Bradford LS, Rauh-Hain JA, Schorge J, Birrer MJ, Dizon DS (2015). Advances in the management of recurrent endometrial cancer. Am. J. Clin. Oncol..

[8] Bleijs M, van de Wetering M, Clevers H, Drost J (2019). Xenograft and organoid model systems in cancer research. EMBO J..

[9] Paul SM (2010). How to improve R&D productivity: the pharmaceutical industry’s grand challenge. Nat. Rev. Drug Discov..

[10] Ben-David U (2018). Genetic and transcriptional evolution alters cancer cell line drug response. Nature.

[11] Korch C (2012). DNA profiling analysis of endometrial and ovarian cell lines reveals misidentification, redundancy and contamination. Gynecol. Oncol..

[12] Van Nyen, T., Moiola, C. P., Colas, E., Annibali, D. & Amant, F. Modeling endometrial cancer: past, present, and future. *Int. J. Mol. Sci.* **19**, 2348 (2018). 10.3390/ijms19082348 PMC6121384 30096949

[13] Hoffman RM (2015). Patient-derived orthotopic xenografts: better mimic of metastasis than subcutaneous xenografts. Nat. Rev. Cancer.

[14] Haldorsen IS (2015). Multimodal imaging of orthotopic mouse model of endometrial carcinoma. PLoS One.

[15] Fonnes T (2018). Asparaginase-like protein 1 expression in curettage independently predicts lymph node metastasis in endometrial carcinoma: a multicentre study. BJOG.

[16] Cabrera S (2012). Generation and characterization of orthotopic murine models for endometrial cancer. Clin. Exp. Metastasis.

[17] Doll A (2009). An orthotopic endometrial cancer mouse model demonstrates a role for RUNX1 in distant metastasis. Int. J. Cancer.

[18] Konings, G. F. et al. Development of an image-guided orthotopic xenograft mouse model of endometrial cancer with controllable estrogen exposure. *Int. J. Mol. Sci.* **19**, 2547 (2018). 10.3390/ijms19092547 PMC6165149 30154339

[19] Schutgens F, Clevers H (2020). Human organoids: tools for understanding biology and treating diseases. Annu. Rev. Pathol..

[20] Sachs N (2018). A living biobank of breast cancer organoids captures disease heterogeneity. Cell.

[21] van de Wetering M (2015). Prospective derivation of a living organoid biobank of colorectal cancer patients. Cell.

[22] Kopper O (2019). An organoid platform for ovarian cancer captures intra- and interpatient heterogeneity. Nat. Med..

[23] Yao Y (2020). Patient-derived organoids predict chemoradiation responses of locally advanced rectal cancer. Cell Stem Cell.

[24] Tiriac H (2018). Organoid profiling identifies common responders to chemotherapy in pancreatic cancer. Cancer Discov.

[25] Ooft, S. N. et al. Patient-derived organoids can predict response to chemotherapy in metastatic colorectal cancer patients. *Sci. Transl. Med.* **11**, eaay2574 (2019). 10.1126/scitranslmed.aay2574 31597751

[26] Vlachogiannis G (2018). Patient-derived organoids model treatment response of metastatic gastrointestinal cancers. Science.

[27] Pauli C (2017). Personalized in vitro and in vivo cancer models to guide precision medicine. Cancer Discov..

[28] Boretto M (2019). Patient-derived organoids from endometrial disease capture clinical heterogeneity and are amenable to drug screening. Nat. Cell. Biol..

[29] Moiola, C. P. et al. Patient-derived xenograft models for endometrial cancer research. *Int. J. Mol. Sci.* **19**, 2431 (2018). 10.3390/ijms19082431 PMC6121639 30126113

[30] Fonnes, T. et al. Near-infrared fluorescent imaging for monitoring of treatment response in endometrial carcinoma patient-derived xenograft models. *Cancers (Basel)* **12**, 370 (2020). 10.3390/cancers12020370 PMC7072497 32041116

[31] Tangen IL (2017). Expression of L1CAM in curettage or high L1CAM level in preoperative blood samples predicts lymph node metastases and poor outcome in endometrial cancer patients. Br. J.Cancer.

[32] Kobel M (2019). Interpretation of P53 immunohistochemistry in endometrial carcinomas: toward increased reproducibility. Int. J. Gynecol. Pathol..

[33] Levine JH (2015). Data-driven phenotypic dissection of AML reveals progenitor-like cells that correlate with prognosis. Cell.

[34] Liston DR, Davis M (2017). Clinically relevant concentrations of anticancer drugs: a guide for nonclinical studies. Clin. Cancer Res..

[35] Helland O (2014). First in-mouse development and application of a surgically relevant xenograft model of ovarian carcinoma. PLoS One.

[36] Shen R, Seshan VE (2016). FACETS: allele-specific copy number and clonal heterogeneity analysis tool for high-throughput DNA sequencing. Nucleic. Acids Res..

[37] Leon-Castillo A (2020). Interpretation of somatic POLE mutations in endometrial carcinoma. J. Pathol..

[38] Krakstad C (2012). Loss of GPER identifies new targets for therapy among a subgroup of ERalpha-positive endometrial cancer patients with poor outcome. Br. J. Cancer.

[39] Subramanian A (2017). A next generation connectivity map: L1000 platform and the first 1,000,000 profiles. Cell.

[40] Turco MY (2017). Long-term, hormone-responsive organoid cultures of human endometrium in a chemically defined medium. Nat Cell Biol.

[41] Talhouk A (2015). A clinically applicable molecular-based classification for endometrial cancers. Br. J. Cancer.

[42] Gibson WJ (2016). The genomic landscape and evolution of endometrial carcinoma progression and abdominopelvic metastasis. Nat. Genet..

[43] Clevers H, Tuveson DA (2019). Organoid models for cancer research. Annu. Rev. Canc. Biol..

[44] Krieger TG (2020). Modeling glioblastoma invasion using human brain organoids and single-cell transcriptomics. Neuro. Oncol..

[45] Rosenbluth JM (2020). Organoid cultures from normal and cancer-prone human breast tissues preserve complex epithelial lineages. Nat. Commun..

[46] Tangen IL (2014). Loss of progesterone receptor links to high proliferation and increases from primary to metastatic endometrial cancer lesions. Eur. J. Cancer.

[47] Wik E (2013). Lack of estrogen receptor-alpha is associated with epithelial-mesenchymal transition and PI3K alterations in endometrial carcinoma. Clin. Cancer Res..

[48] Alexandrov LB (2020). The repertoire of mutational signatures in human cancer. Nature.

[49] Alexandrov LB (2013). Signatures of mutational processes in human cancer. Nature.

[50] Yan T (2019). Multi-region sequencing unveils novel actionable targets and spatial heterogeneity in esophageal squamous cell carcinoma. Nat. Commun..

[51] Jamal-Hanjani M (2017). Tracking the evolution of non-small-cell lung cancer. N. Engl. J. Med..

[52] Warrick JI (2019). Intratumoral heterogeneity of bladder cancer by molecular subtypes and histologic variants. Eur. Urol..

[53] Ramon YCS (2020). Clinical implications of intratumor heterogeneity: challenges and opportunities. J. Mol. Med. (Berl.).

[54] Clarke MA (2018). Association of endometrial cancer risk with postmenopausal bleeding in women: a systematic review and meta-analysis. JAMA. Intern.Med..

[55] Fleming GF (2007). Systemic chemotherapy for uterine carcinoma: metastatic and adjuvant. J. Clin. Oncol..

[56] Pectasides D (2008). Carboplatin and paclitaxel in advanced or metastatic endometrial cancer. Gynecol. Oncol..

[57] Arora, S. et al. FDA approval summary: pembrolizumab plus lenvatinib for endometrial carcinoma, a collaborative international review under project orbis. *Clin. Cancer Res.* **26**, 5062–5067 (2020). 10.1158/1078-0432.CCR-19-3979 32295834

[58] Abdul Aziz NA (2016). A 19-Gene expression signature as a predictor of survival in colorectal cancer. BMC Med. Genomics.

[59] Lim SY, Yuzhalin AE, Gordon-Weeks AN, Muschel RJ (2016). Tumor-infiltrating monocytes/macrophages promote tumor invasion and migration by upregulating S100A8 and S100A9 expression in cancer cells. Oncogene.

[60] Wang T (2020). COL4A1 promotes the growth and metastasis of hepatocellular carcinoma cells by activating FAK-Src signaling. J. Exp. Clin. Cancer Res..

[61] van der Velden DL (2019). The drug rediscovery protocol facilitates the expanded use of existing anticancer drugs. Nature.

[62] Gao D (2014). Organoid cultures derived from patients with advanced prostate cancer. Cell.

[63] Maenhoudt N (2020). Developing organoids from ovarian cancer as experimental and preclinical models. Stem Cell Reports.

[64] Fujii M (2016). A colorectal tumor organoid library demonstrates progressive loss of niche factor requirements during tumorigenesis. Cell Stem Cell.

[65] Sachs, N. et al. Long-term expanding human airway organoids for disease modeling. *EMBO J.* **38**, e100300 (2019). 10.15252/embj.2018100300 PMC6376275 30643021

[66] Yuki K, Cheng N, Nakano M, Kuo CJ (2020). Organoid models of tumor immunology. Trends Immunol..

[67] Kusonmano K (2018). Identification of highly connected and differentially expressed gene subnetworks in metastasizing endometrial cancer. PLoS One.

