# Author Correction: Patient-derived organoids reflect the genetic profile of endometrial tumors and predict patient prognosis

**DOI:** 10.1038/s43856-021-00022-2

**Published:** 2021-08-10

**Authors:** Hege F. Berg, Marta Espevold Hjelmeland, Hilde Lien, Heidi Espedal, Tina Fonnes, Aashish Srivastava, Tomasz Stokowy, Elin Strand, Olivera Bozickovic, Ingunn M. Stefansson, Line Bjørge, Jone Trovik, Ingfrid S. Haldorsen, Erling A. Hoivik, Camilla Krakstad

**Affiliations:** 1 Department of Clinical Science, Centre for Cancer Biomarkers, UiB, Bergen, Norway; 2 Department of Gynecology and Obstetrics, Haukeland University Hospital, Bergen, Norway; 3 Section of Radiology, Department of Clinical Medicine, UiB, Bergen, Norway; 4 Department of Radiology, Mohn Medical Imaging and Visualization Centre, Haukeland University Hospital, Bergen, Norway; 5 Section of Bioinformatics, Clinical Laboratory, Haukeland University Hospital, Bergen, Norway; 6 Genomics Core Facility, Department of Clinical Science, UiB, Bergen, Norway; 7 Section for Pathology, Department of Clinical Medicine, Haukeland University Hospital, Bergen, Norway

**Keywords:** Endometrial cancer, Cancer genomics, Chemotherapy, Cancer models, Cancer models

Correction to: *Communications Medicine* 10.1038/s43856-021-00019-x, published online 30 July 2021.

The original version of this Article contained an error in the spelling of the author Tomasz Stokowy, which was incorrectly given as Tomasz Stokowsky. This has now been corrected in both the PDF and HTML versions of the Article.

