# Supplementary material for: Patient-derived organoids reflect the genetic profile of endometrial tumors and predict patient prognosis
Source: Commun Med (Lond). 2021 Jul 30;1:20. doi: 10.1038/s43856-021-00019-x (PMC9053236; doi:10.1038/s43856-021-00019-x)
Supplement: Supplementary file 1 — Supplementary Information [file 43856_2021_19_MOESM1_ESM.pdf]

Supplementary Table 1. IHC antibody information

Antibody	Supplier	Catalogue number	Dilution	Incubation	HIER buffer
ERα	DAKO	M7047	1:50	30 min	pH 9
PR	DAKO	M3569	1:150	30 min	pH 9
p53	DAKO	M7001	1:1000	30 min	pH 9
EpCAM	Cell signaling	D9S3P	1:200	60 min	pH 6
L1CAM	BioLegend	SIG-3911	1:100	60 min	pH 9
PTEN	Cell Signaling	9188	1:100	4ºC overnight	pH 6
ARID1A	Abcam	ab182560	1:2000	60 min	pH 6
MSH6	Leica Biosystems	MSH6-L-CE	1:25	60 min	pH 9
MSH2	Leica Biosystems	MSH2-L-CE	1:50	30 min	pH 6
PMS2	Leica Biosystems	PMS2-L-CE	1:25	60 min	pH 9
MLH1	Leica Biosystems	MLH1-L-CE	1:100	30 min	pH 9
Ki67	Abcam	Ab16667	1:100	60 min	pH 6

Supplementary Table 2. IMC antibody information

Mass isotope	Target	Clone	Staining Conc[a]	Supplier	Catalogue no
**141 Pr**	α-SMA	1A4	2.5	Fluidigm	3141017D
**143 Nd**	Vimentin	D21H3	5.0	Fluidigm	3143027D
**144 Nd**	EpCAM	9C4	6.7	Fluidigm	3144026D
**146 Nd**	CD16	EPR16784	5.0	Fluidigm	3146020D
**148 Nd**	Pan-cytokeratin	AE1/AE3	10.0	Fluidigm	3148022D
**150 Nd**	PD-L1	E1L3N	5.0	Fluidigm	3150031D
**151 Eu**	CD31	EPR3094	5.0	Fluidigm	3151025D
**152 Sm**	CD45	CD45-2B11	6.7	Fluidigm	3152016D
**154 Sm**	ER	SP1	5.0	Abcam	ab187260
**156 Gd**	CD4	EPR6115	5.0	Fluidigm	3156033D
**158 Gd**	E-Cadherin	24E10	5.0	Fluidigm	3158029D
**159 Tb**	CD68	KP1	5.0	Fluidigm	3159035D
**161 Dy**	CD20	H1	1.3	Fluidigm	3161029D
**162 Dy**	CD8a	D8A8Y	5.0	Fluidigm	3162035D
**163 Dy**	VEGF	G153-694	5.0	Fluidigm	3163028D
**164 Dy**	PDGFRα	EPR22059-270	10.0	Abcam	ab234965
**165 Ho**	β-catenin	D13A1	2.5	Fluidigm	3165032D
**167 Er**	Podoplanin	D2-40	5.0	Biolegend	916606
**168 Er**	Ki-67	B56	5.0	Fluidigm	3168022D
**169 Tm**	Collagen type I	Polyclonal	5.0	Fluidigm	3169023D
**170 Er**	CD3	C-terminal	10.0	Fluidigm	3170019D
**171 Yb**	pERK1/2	D13.14.E4	10.0	Fluidigm	3171021D
**173 Yb**	PR	SP2	6.7	Abcam	ab239793
**175 Lu**	pS6	N7-548	6.7	Fluidigm	3175031D
**176 Yb**	HH3	D1H2	1.3	Fluidigm	3176023D
**191/193 Ir**	Iridium	-	1:300	Fluidigm	201192A

[a] μg/ml

Supplementary Table 3. PCR primers for sequencing of POLE exon 9, 11, 13 and 14.

Exon	Forward PCR primer (5`-3`)*	Reverse PCR primer (5`-3`)*	PCR size
9	**TGTAAAACGACGGCCAGT**GCCTAATGGGGAGTTTAGAGC	**CAGGAAACAGCTATGACC**TACTTCCCAGAAGCCACCTG	219
11	**TGTAAAACGACGGCCAGT**GAGAAAGAGCAGACCTCTGAC	**CAGGAAACAGCTATGACC**CCAGTTACTCATAGAGAAGACACAGA	193
13	**TGTAAAACGACGGCCAGT**TCTGTTCTCATTCTCCTTCCAG	**CAGGAAACAGCTATGACC**CGGGATGTGGATTACGTG	210
14	**TGTAAAACGACGGCCAGT**TCTGGCGTTCTCTCCTCAG	**CAGGAAACAGCTATGACC**CGACAGGACAGATAATGCTCA	191

* Universal primers (bold) are incorporated into forward and reverse primers for subsequent Sanger sequencing.

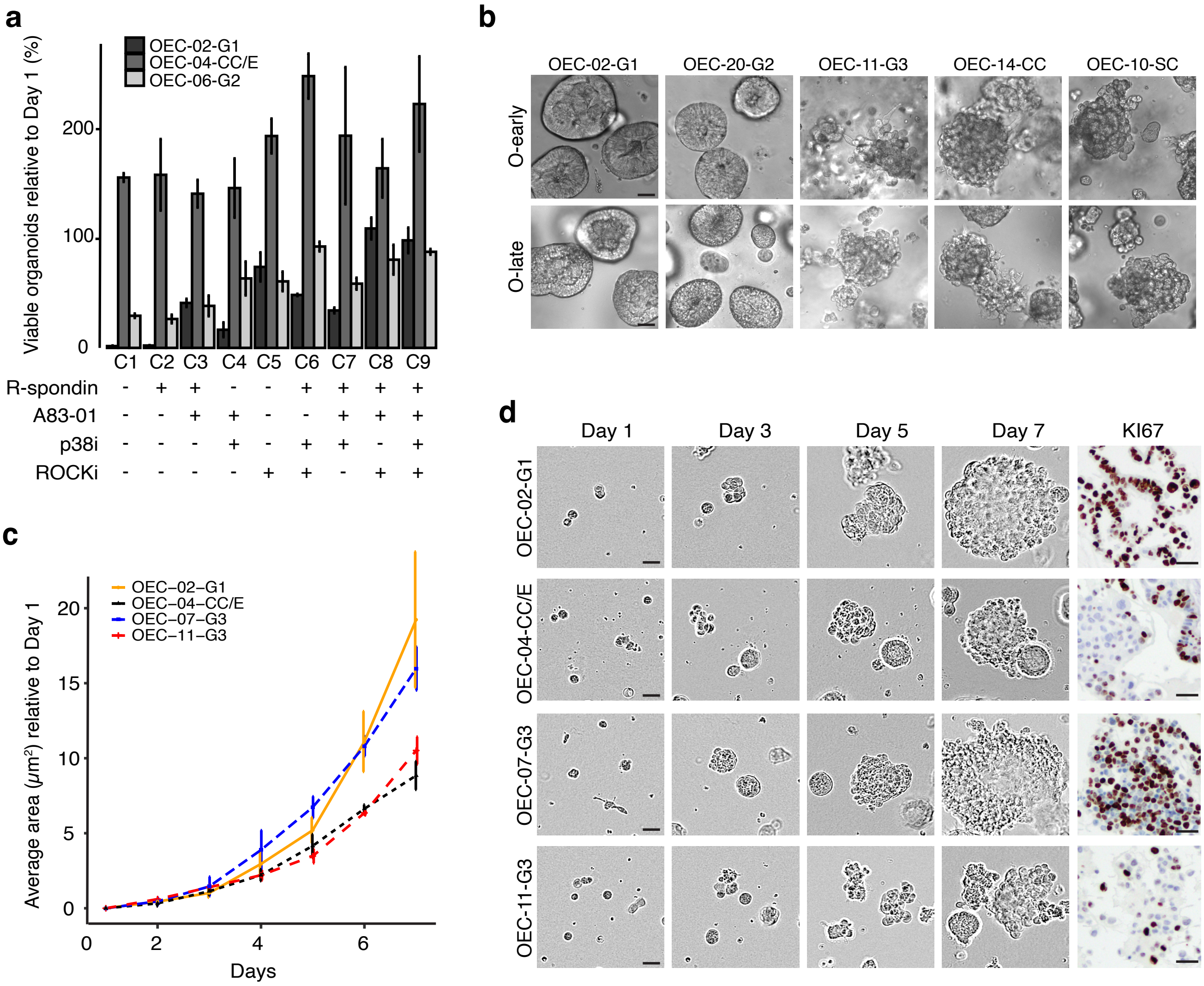

Supplementary Figure 1. Modified medium allows long-term expansion of EC organoids. Growth rates differ between the cultures.

a Medium whithdrawal experiment showing viable organoids in three different organoid cultures after eight days of culture with nine different medium conditions (C1-C9). Organoids were seeded as small cell aggregates and number of viable organoids (≥ 2 cells in cluster) at Day 8 was normalized to counts at Day 1. Error bars represent SD of three technical replicates. **b** Representative brightfield images of early- and late-passaged organoids derived from different subtypes of endometrial tumors. **c** Average phase object area (mm^2) of selected organoid cultures at day 2-7 normalized to Day 1. Phase object area for each organoid culture was calculated using the IncuCyte ZOOM software. Error bars represent SD of two technical replicates. **d** Representative brightfield images of organoid cultures at 1, 3, 5 and 7 days after seeding. Organoids were seeded as small aggregates of cells in Matrigel coated wells in EC-ExM/5% Matrigel. Right panel depicts Ki67 immunohistochemical staining of corresponding organoids. Scale bars = 20 µm.

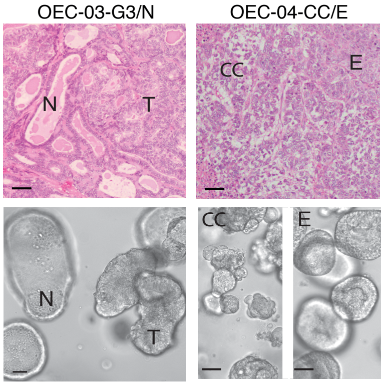

Supplementary Figure 2. Mixed tumor histology is retained in the organoid cultures. Representative H&E stained sections of patient tissue and brightfield images of corresponding mixed-morphology organoid cultures. Subclones are indicated with T=tumor, N=normal, CC=clear cell, E=endometrioid. Scale bar = 50 µm.

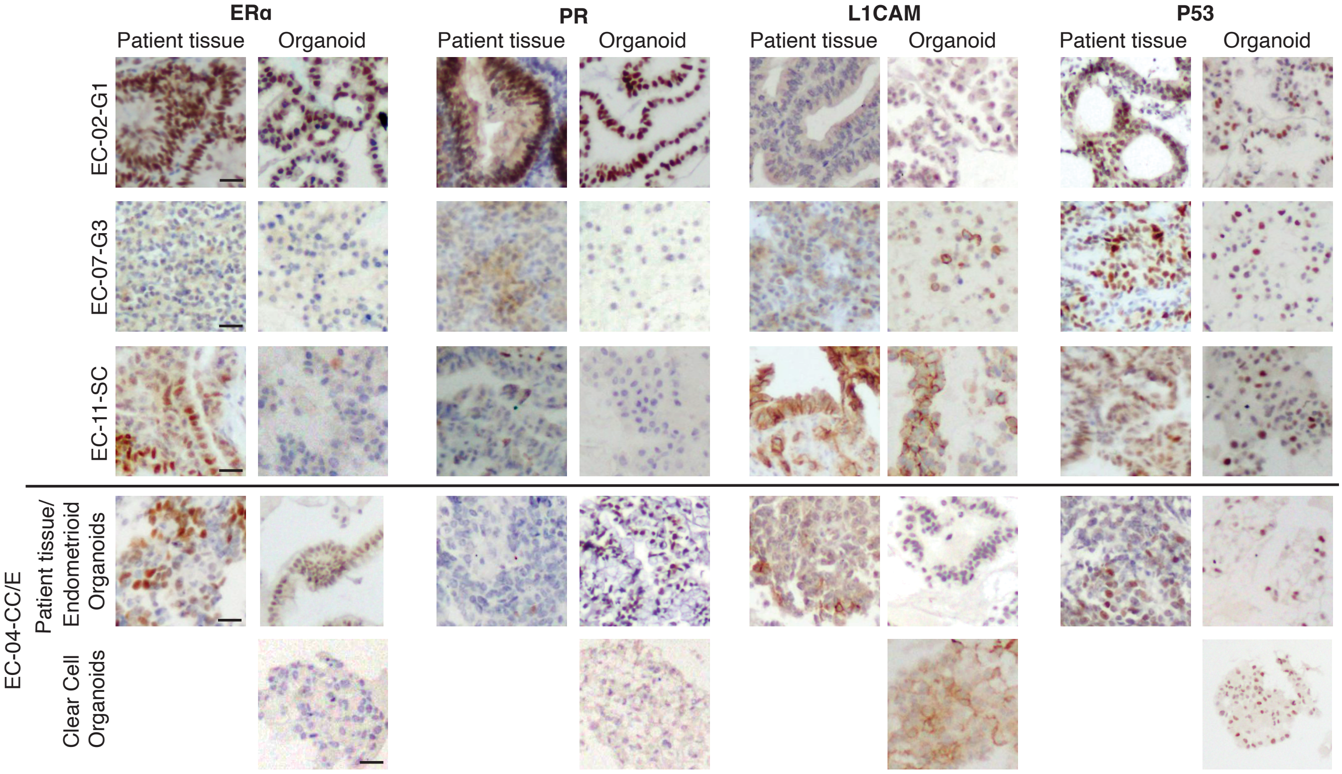

Supplementary Figure 3. Patient tissue biomarker expression is retained in the organoids. Representative images of selected primary tumor-organoid pairs showing immunohistochemical staining of ER, PR, L1CAM and p53. In the lower panel, stained sections of the clear cell component of EC-04-CC/E patient tissue are depicted as well as the subcloned pure endometrioid and clear cell organoid cultures. Scale bars = 20 µm.

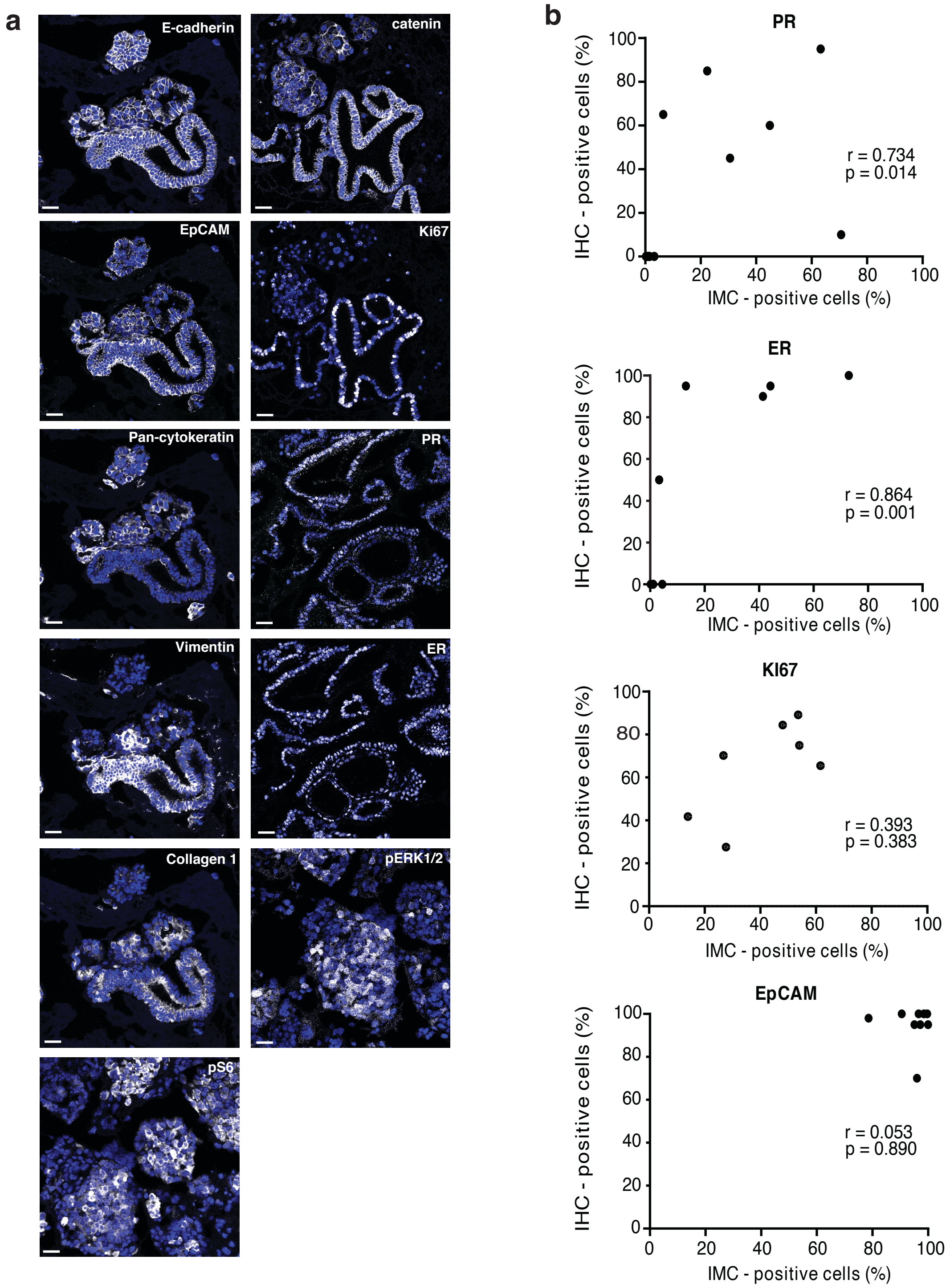

Supplementary Figure 4. Marker expression measured by imaging mass cytometry (IMC) and immunohistochemistry (IHC) is similar.

a Example images from IMC depicting positive staining of the tumor cell markers Vimentin, Pan-cytokeratin, EpCAM, Collagen I, PR, ER, β-catenin, Ki67, pERK1/2, pS6 and E-cadherin. Scale bar = 20 μ m. **b** For IMC, single cells positive for a marker of interest were quantified by manually gating single cells in HistoCAT. Percent positive cells identified by IMC was plotted against percent positive cells determined by manually scoring of IHC stained sections. The concordance between the two methods were calculated using Spearman correlation.

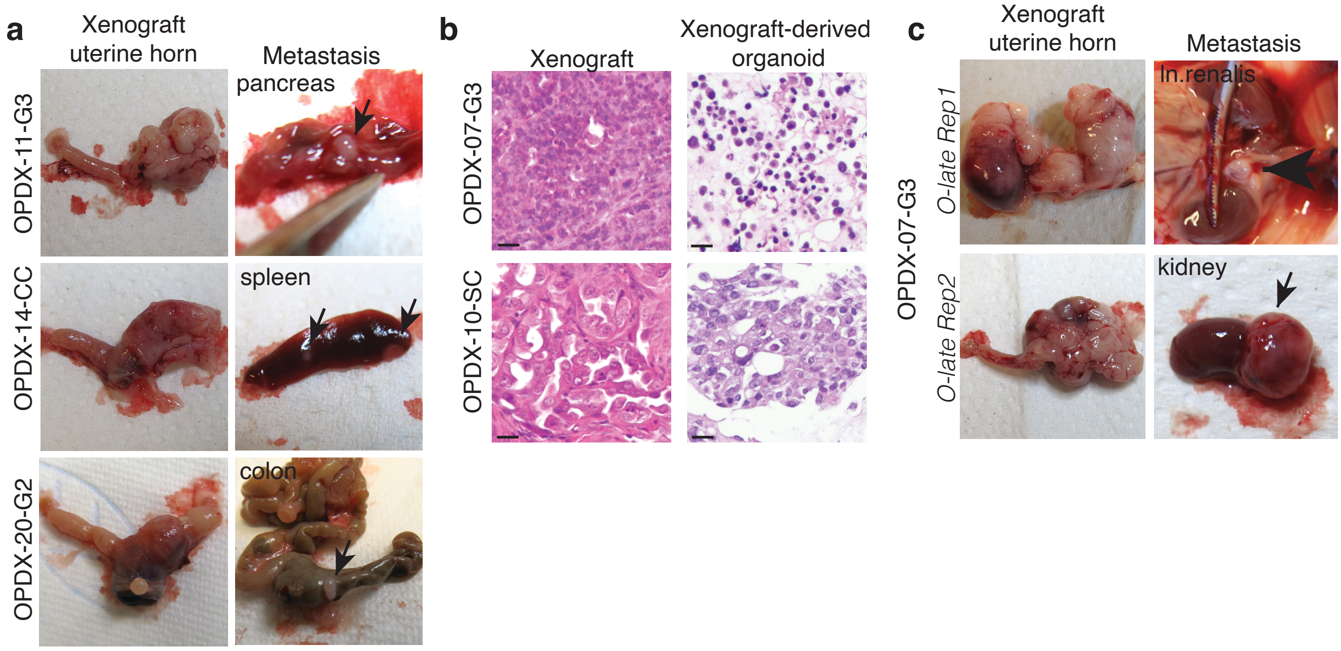

Supplementary Figure 5. Orthotopically implanted organoids metastasize to lymph nodes and distant organs.

a Representative O-PDX models with large tumor mass in left uterine horn and metastases to distant organs. **b** Representative H&E stained sections of uterine xenografts and corresponding xenograft-derived organoids. **c** O-PDX model generated by implanting late-passaged organoids. Mice developed large uterine tumors with metastases to either renal lymph nodes or kidney. Scale bar = 20 µm.

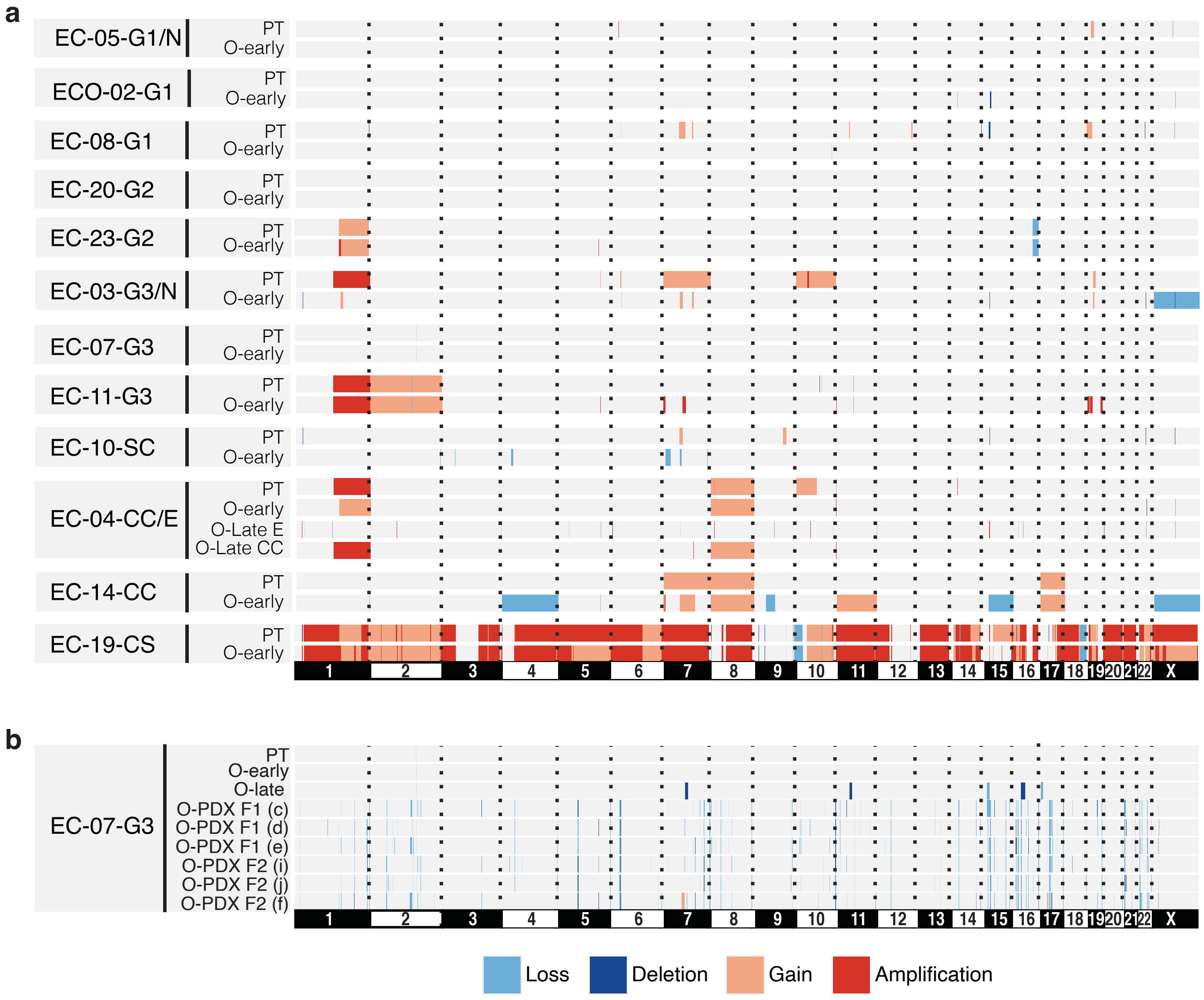

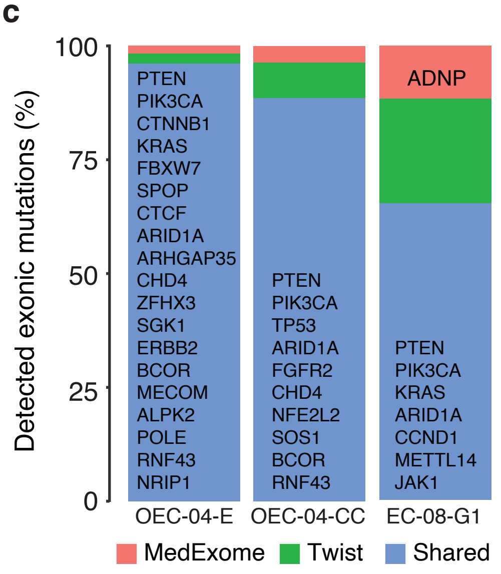

Supplementary Figure 6. Copy number variations (CNVs) of paired samples. Comparison between Twist Human Core Exome versus SeqCap EZ MedExome capture systems.
a Panel depicting loss/deletions (blue) and gains/amplifications (red) in primary tumor and corresponding organoid. CNVs were generated from WES data using FACETS. **b** CNVs in paired samples from EC-07-G3, including multiple O-PDX samples. A detailed description of sample origin is given in Figure 5B and D. **c** Three samples were subjected to both MedExome and Twist library preparation and mutations in the exonic regions were selected for comparative analysis. Detected EC-specific driver mutations are listed within the bars.

Supplementary Table 4. Differentially expressed genes in cluster 2 versus cluster 1

Transcript	Log2 fold change	FDR (q-value)
S100A9	7.80	<0.001
SORCS2	7.03	<0.001
POU3F3	7.24	<0.001
COL4A2	6.97	<0.001
COL4A1	6.55	<0.001
RNF182	5.96	<0.001
PRTG	7.78	<0.001
SIRPA	6.70	<0.001
SAMD5*	7.47	<0.001
MMP26	-7.87	<0.001
SCGB2A1	-8.09	<0.001
SCGB1D2	-7.38	<0.001
PGR	-8.88	<0.001
KIAA1324	-6.87	<0.001
CST1	-7.71	<0.001
SCGB2A2	-7.90	<0.001
PKHD1L1	-6.82	<0.001
ESR1	-8.48	<0.001
SERPINA6	-6.42	<0.001
CAPN6	-6.62	<0.001
IHH*	-8.09	<0.001
RP11-6E9.4*	-7.30	<0.001
RP11-474D1.3*	-6.48	<0.001

*Genes excluded from 19-gene signature (see Methods section)

Supplementary Table 5. Signature score associates with aggressive features of endometrial cancer.

	Low risk sign.	High risk sign.	
	n (%)	n(%)	P-value[d]
**Number of patients**	148	108	
**Age, median**			**0.002**
<67	90 (66.7)	45 (33.3)	
≥67	58 (47.9)	63 (52.1)	
**Histologic type and grade [a]**			**<0.001**
Endometrioid grade 1-2	120 (81.1)	28 (18.9)	
Endometrioid grade 3	18 (36.0)	32 (64.0)	
Non-endometrioid	5 (9.4)	48 (90.6)	
**FIGO stage**			**<0.001**
I	122 (66.3)	62 (33.7)	
II	11 (55.0)	9 (45.0)	
III	13 (36.1)	23 (63.9)	
IV	2 (12.5)	14 (87.5)	
**Metastatic lymph nodes[b]**			**<0.001**
Yes	104 (61.9)	64 (38.1)	
No	9 (27.3)	24 (72.7)	
**Myometrial infiltration[c]**			**0.012**
<50%	85 (65.9)	44 (34.1)	
>50%	63 (50.4)	62 (49.6)	

Abbreviations: FIGO: International Federation of Gynaecology and Obstetrics.
[a]Data missing for 5 patients, [b]Data missing for 55 patients, [c]Data missing for 2 patients, [d]Pearson chi-square test.

Supplementary Table 6. Signature score in relation to clinicopathological factors in the L1000 and TCGA transcriptomic EC datasets.

	L1000			TCGA		
	**Low risk sign.**	**High risk sign.**	P-value[e]	**Low risk sign.**	**High risk sign.**	P-value[c]
**Number of patients**	230	150		293	213	
**Age, median[a]**			**0.001**			**0.001**
<64	131 (68.6)	60 (31.4)		177 (64.8)	96 (35.2)	
≥64	99 (52.4)	90 (47.6)		114 (49.6)	116 (50.4)	
**Histologic subtype**			**<0.001**			**<0.001**
Endometrioid grade 1-2	203 (82.2)	44 (17.8)		179 (84.0)	34 (16.0)	
Endometrioid grade 3	18 (33.3)	36 (66.7)		100 (54.3)	84 (45.7)	
Non-endometrioid	6 (7.9)	70 (92.1)		14 (12.8)	95 (87.2)	
**FIGO stage**			**0.001**			
I	192 (64.6)	105 (35.4)				
II	18 (56.3)	14 (43.8)				
III	18 (47.4)	20 (52.6)				
IV	2 (15.4)	11 (84.6)				
**Metastatic LN[b]**			**0.004**			
Yes	175 (62.3)	106 (37.7)				
No	13 (37.1)	22 (62.9)				
**Myometrial infiltration**			0.079			
<50%	145 (64.2)	81 (35.8)				
≥50%	85 (55.2)	69 (44.8)				

Abbreviations: FIGO: International Federation of Gynaecology and Obstetrics.
[a]Data missing for 3 patients in the TCGA cohort, [b]LN: lymph nodes: Data missing for 64 patients in the L1000 cohort, [c]Pearson chi-square test.
