# Supplementary material for: Patient-derived organoids reflect the genetic profile of endometrial tumors and predict patient prognosis
Source: Commun Med (Lond). 2021 Jul 30;1:20. doi: 10.1038/s43856-021-00019-x (PMC9053236; doi:10.1038/s43856-021-00019-x)
Supplement: Supplementary file 4 — Description of Additional Supplementary Files [file 43856_2021_19_MOESM4_ESM.pdf]

Description of Additional Supplementary Files

File name: Supplementary Data 1

Description: List of all received tissue samples**.**

File name: Supplementary Data 2

Description: All source data underlying the graphs and charts.
