# Supplementary material for: Patient-derived organoids reflect the genetic profile of endometrial tumors and predict patient prognosis
Source: Commun Med (Lond). 2021 Jul 30;1:20. doi: 10.1038/s43856-021-00019-x (PMC9053236; doi:10.1038/s43856-021-00019-x)
Supplement: Supplementary file 5 — Reporting Summary [file 43856_2021_19_MOESM5_ESM.pdf]

nature research | reporting summary

nature research

Corresponding author(s): COMMSMED-21-0054C

Last updated by author(s): Jun 25, 2021

Reporting Summary

Nature Research wishes to improve the reproducibility of the work that we publish. This form provides structure for consistency and transparency in reporting. For further information on Nature Research policies, see our Editorial Policies and the Editorial Policy Checklist.

Statistics

For all statistical analyses, confirm that the following items are present in the figure legend, table legend, main text, or Methods section.

n/a	Confirmed	
☐	☒	The exact sample size (*n*) for each experimental group/condition, given as a discrete number and unit of measurement
☐	☒	A statement on whether measurements were taken from distinct samples or whether the same sample was measured repeatedly
☐	☒	The statistical test(s) used AND whether they are one- or two-sided<br>*Only common tests should be described solely by name; describe more complex techniques in the Methods section.*
☒	☐	A description of all covariates tested
☐	☒	A description of any assumptions or corrections, such as tests of normality and adjustment for multiple comparisons
☐	☒	A full description of the statistical parameters including central tendency (e.g. means) or other basic estimates (e.g. regression coefficient) AND variation (e.g. standard deviation) or associated estimates of uncertainty (e.g. confidence intervals)
☐	☒	For null hypothesis testing, the test statistic (e.g. *F*, *t*, *r*) with confidence intervals, effect sizes, degrees of freedom and *P* value noted<br>*Give P values as exact values whenever suitable.*
☒	☐	For Bayesian analysis, information on the choice of priors and Markov chain Monte Carlo settings
☐	☒	For hierarchical and complex designs, identification of the appropriate level for tests and full reporting of outcomes
☒	☐	Estimates of effect sizes (e.g. Cohen's *d*, Pearson's *r*), indicating how they were calculated

Our web collection on statistics for biologists contains articles on many of the points above.

Software and code

Policy information about availability of computer code

Data collection	No software was used.
Data analysis	For processing IMC images: https://github.com/BodenmillerGroup/ImcSegmentationPipeline<br>Publicly-available software used in this study includes: Maftools, FACETS, MCD Viewer, Chromas, bwa-mem, samtools, Picard, bedtools, Varscan, Annovar, hisat, Feature Counts, DESeq2, JExpress, SPSS, GraphPad/Prism. The tools and their versions are reported in detail in material and methods section.

For manuscripts utilizing custom algorithms or software that are central to the research but not yet described in published literature, software must be made available to editors and reviewers. We strongly encourage code deposition in a community repository (e.g. GitHub). See the Nature Research guidelines for submitting code & software for further information.

Data

Policy information about availability of data

All manuscripts must include a data availability statement. This statement should provide the following information, where applicable:

- Accession codes, unique identifiers, or web links for publicly available datasets
- A list of figures that have associated raw data
- A description of any restrictions on data availability

Source data used to generate graphs and charts are included in Supplementary Data 2. Primer sequences used for POLE sequencing are included in the Supplementary Table 3. Transcriptomic datasets are available at ArrayExpress: RNAseq dataset [E-MTAB-10664], Agilent Microarray dataset [E-MTAB-5017], L1000 dataset [E-MTAB-10668]. The TCGA dataset (PanCancer Atlas) can be accessed via cBioPortal (https://www.cbioportal.org/datasets). Patient consent do not allow for deposition of WES data in public/controlled access repositories. Interested researchers should contact C.K. (camilla.krakstad@uib.no) to inquire about access; requests for noncommercial academic use will be considered and require ethics review.

Field-specific reporting

Please select the one below that is the best fit for your research. If you are not sure, read the appropriate sections before making your selection.

☒ Life sciences ☐ Behavioural & social sciences ☐ Ecological, evolutionary & environmental sciences

For a reference copy of the document with all sections, see nature.com/documents/nr-reporting-summary-flat.pdf

Life sciences study design

All studies must disclose on these points even when the disclosure is negative.

Sample size	For O-PDX treatment experiment: >10 mice in each group were considered enough to evaluate treatment effect.<br>For organoid growth rate and drug testing: 2 ≥ independent experiments were considered enough to demonstrate inter-organoid heterogeneity in drug response.
Data exclusions	For hierarchical clustering analysis: transcripts with expression levels < 5 were considered noice. Sample mean log2 value < 5 were thus excluded from downstream analyses.
Replication	Orthotopic implantation of organoids was repeated for selected models. All attempts of replication were successful.
Randomization	Animals were assigned randomly to experimental groups.
Blinding	Scoring of immunohistochemical staining was performed blinded.

Reporting for specific materials, systems and methods

We require information from authors about some types of materials, experimental systems and methods used in many studies. Here, indicate whether each material, system or method listed is relevant to your study. If you are not sure if a list item applies to your research, read the appropriate section before selecting a response.

Materials & experimental systems

n/a	Involved in the study	
☐	☒	Antibodies
☒	☐	Eukaryotic cell lines
☒	☐	Palaeontology and archaeology
☐	☒	Animals and other organisms
☐	☒	Human research participants
☒	☐	Clinical data
☒	☐	Dual use research of concern

Methods

n/a	Involved in the study	
☒	☐	ChIP-seq
☒	☐	Flow cytometry
☒	☐	MRI-based neuroimaging

Antibodies

Antibodies used	Antibody/Manufracturer/Catalogue number/Clone/Lot: ERα/DAKO/M7047/1D5/00067115, PR/DAKO/M3569/PgR 636/10046506, p53/DAKO/M7001/DO-7/20057047, EpCAM/CellSignalling/14452S/D9S3P/unknown, L1CAM/Biolegend/SIG-3911/14.10/826701, PTEN/CellSignalling/9188/D4.3/6, ARID1A/Abcam/ab182560/EPR13501-73/gr3240244-2, MSH6/Leica/MSH6-L-CE/PU29/6075676, MSH2/Leica/MSH2-CE-S/25D12/6065019, PMS2/Leica/PMS2-L-CE/M0R4G/6067483, MLH1/Leica/MLH1-L-CE/ES05/6063040, Ki67/ab16667/SP6.
Validation	All antibodies were used according to the manufacturers recommendations. Antibody concentrations and antigen retrieval pH were optimized for each antibody. Details on staining conditions are included in material and methods section.

Animals and other organisms

Policy information about studies involving animals; ARRIVE guidelines recommended for reporting animal research

Laboratory animals	NOD.Cg-Prkdc scid IL2rg tm1WjI/SzJ (NSG) female mice were used for all animal experiments.
Wild animals	Study did not involve wild animals.
Field-collected samples	Study did not involve samples collected from the field.
Ethics oversight	Ethical approval was granted from the Norwegian Food Safety Authority.

Note that full information on the approval of the study protocol must also be provided in the manuscript.

Human research participants

Policy information about studies involving human research participants

Population characteristics	Donor characteristics for all organoid models are described in detail in the manuscript, table 1, except age to minimize risk of identifying patients. A population based endometrial cancer cohort was used for validation. Metadata and clinico-pathological data including histological grade, type, FIGO stage are described for the validation cohort in the manuscript.
Recruitment	Human specimens were prospectively collected from consenting patients treated for endometrial cancer at our institution.
Ethics oversight	Norwegian regional committees for medical and health research ethics

Note that full information on the approval of the study protocol must also be provided in the manuscript.
